# SUMOylation of the lysine-less tumor suppressor p14ARF counters ubiquitylation-dependent degradation

**DOI:** 10.1038/s41419-025-07854-z

**Published:** 2025-07-12

**Authors:** Ahmed El Motiam, Yanis H. Bouzaher, Haifen Chen, Rocío Seoane, Santiago Vidal, María Blanquer, Rocío M. Tolosa, Beatriz Rodríguez-Lemus, José A. Herrera-Gavilán, Anxo Vidal, Ignacio Palmero, Manuel S. Rodríguez, James D. Sutherland, Rosa Barrio, Dimitris Xirodimas, Manuel Collado, Rod Bremner, Carmen Rivas

**Affiliations:** 1 Centro de Investigación en Medicina Molecular (CIMUS), Universidade de Santiago de Compostela, Instituto de Investigaciones Sanitarias (IDIS), Santiago de Compostela, Spain; 2 Lunenfeld Tanenbaum Research Institute, Mt Sinai Hospital, Sinai Health System, Toronto, ON Canada; 3 Departments of Ophthalmology and Vision Sciences, and Lab Medicine and Pathobiology, University of Toronto, Toronto, ON Canada; 4 Department of Microbiology, Icahn School of Medicine at Mount Sinai, New York, NY USA; 5 Instituto de Investigaciones Biomédicas “Sols-Morreale” CSIC-UAM, Madrid, Spain; 6 Laboratoire de Chimie de Coordination LCC-UPR 8241-CNRS, Toulouse, France; 7 Center for Cooperative Research in Biosciences (CIC bioGUNE), Basque Research and Technology Alliance (BRTA), Derio, Spain; 8 Montpellier Cell Biology Research Center (CRBM), CNRS-UMR 5237 Université de Montpellier, Montpellier, France; 9 Oncology and Immunology Department, Centro Nacional de Biotecnología (CNB)-CSIC, Madrid, Spain; 10 Molecular and Cellular Biology, Centro Nacional de Biotecnología (CNB)-CSIC, Madrid, Spain

**Keywords:** Sumoylation, Ubiquitylation

## Abstract

p14ARF is a lysine-less tumor suppressor that enhances SUMOylation of its interactors. Although p14ARF is known to interact with the E2 SUMO conjugating enzyme UBC9, the link between ARF and SUMOylation is poorly understood and the potential impact of SUMOylation on p14ARF is unknown. Here we show that SUMO2 conjugates to the N-terminus of p14ARF and stabilizes it. Either depleting UBC9 or pharmacologically inhibiting SUMOylation, induces p14ARF degradation. In contrast, blocking ubiquitination or NEDDylation, with TAK-243 or MLN4924/Pevonedistat respectively, increases p14ARF SUMOylation and restores p14ARF levels when SUMOylation is blocked. Treatment with MLN4924 also causes p14ARF-dependent mRNA upregulation of the SUMOylation components SUMO1, SUMO2, and UBC9, globally augmenting SUMOylation. Finally, p14ARF contributes to MLN4924-driven cytotoxicity of prostate cancer cells. Our results provide evidence that, despite lacking lysine, p14ARF is SUMOylated and this modification is critical to counter ubiquitin driven degradation and establishes a new link between inhibition of NEDDylation and SUMOylation.

## Introduction

ARF is a nucleolar protein that exhibits tumor-suppressive functions by stabilizing and stimulating p53 activity. In addition, ARF binds diverse cellular proteins resulting in p53-independent tumor suppressor activities. One of the p53-independent actions of ARF is to promote the conjugation of small ubiquitin-like modifier (SUMO) to interacting proteins such as p53 [1], MDM2 [2], WRN [3], RPL11 [4], NPM [5], and others [6]. The molecular mechanisms involved in ARF-induced SUMOylation are unclear, but it has been proposed that p14ARF, through its interaction with the SUMO-conjugating enzyme UBC9 [6], may facilitate the transfer of SUMO from the E2 complex to ARF-binding proteins. In addition, p19Arf triggers degradation of the SUMO protease SENP3 increasing SENP3 substrate levels [7].

p14ARF levels are regulated at transcriptional and post-transcriptional levels. ARF gene loss and silencing by promoter hyper-methylation or mutation occurs in different cancers [8], and various transcription factors repress p14ARF expression [8, 9]. In contrast, different stimuli induce p14ARF, such as virus infection [10] or oncogenic stress [11–17]. Proteasome-mediated degradation also regulates p14ARF protein, which can occur in a ubiquitin-dependent or -independent manner [18–20]. Ubiquitin conjugation occurs at the p14ARF N-terminus and is promoted by a Cullin-2 RING ligase2 (CRL2) E3 ubiquitin ligase complex [21, 22]. For full activity, the CRL2 complex requires the conjugation of the neural precursor cell expressed developmentally downregulated 8 (NEDD8) protein to Cullin-2. Consequently, inhibition of NEDDylation with MLN4924/Pevonedistat leads to the accumulation of several CRL substrates facilitating its anti-tumor potential. In addition to inducing ubiquitin-dependent degradation of many cellular proteins through activation of CRLs, NEDD8 modification can also promote the stabilization of specific targets [23]. Interestingly, this process can be modulated by conjugation of SUMO which can be promoted by p14ARF [4].

Conjugation of SUMO to specific residues on target proteins, or SUMOylation, requires the E1 SUMO-activating enzyme SAE1/SAE2, the sole SUMO-conjugating E2 enzyme UBC9, and an E3 ligase [24]. However, UBC9 may directly facilitate SUMOylation of the target protein. SUMO conjugation usually occurs at the ΨKxD/E consensus motif where Ψ is an hydrophobic residue, but can also occur on lysines in non-consensus sites [25, 26] and at the N-terminal residue of the substrate protein [27]. UBC9 is a p14ARF interactor [6] and proteomic data identified p14ARF protein as a potential SUMO2 substrate [28]. However, whether SUMO2 modifies this tumor suppressor has not been validated and, more importantly, as p14ARF is a lysine-less protein it is unclear how it might be SUMOylated, and the effect of SUMOylation on p14ARF stability, localization and/or function is unknown.

Here, we validate the SUMO2 modification of p14ARF and demonstrate that SUMOylation significantly increases p14ARF protein stability. We further show that inhibiting NEDDylation with MLN4924 induces the mRNAs of several SUMOylation machinery components, increasing p14ARF SUMOylation and protein levels. Interestingly, MLN4924-induced upregulation of SUMOylation requires p14ARF. Finally, we show that p14ARF contributes to the cytotoxic effect of MLN4924. Taken together, our data support a new interplay between SUMOylation, NEDDylation, and p14ARF with important consequences for the cytotoxic effect of MLN4924.

## Results

### p14ARF protein is modified by SUMO in vitro and in cells

**Fig. 1:**
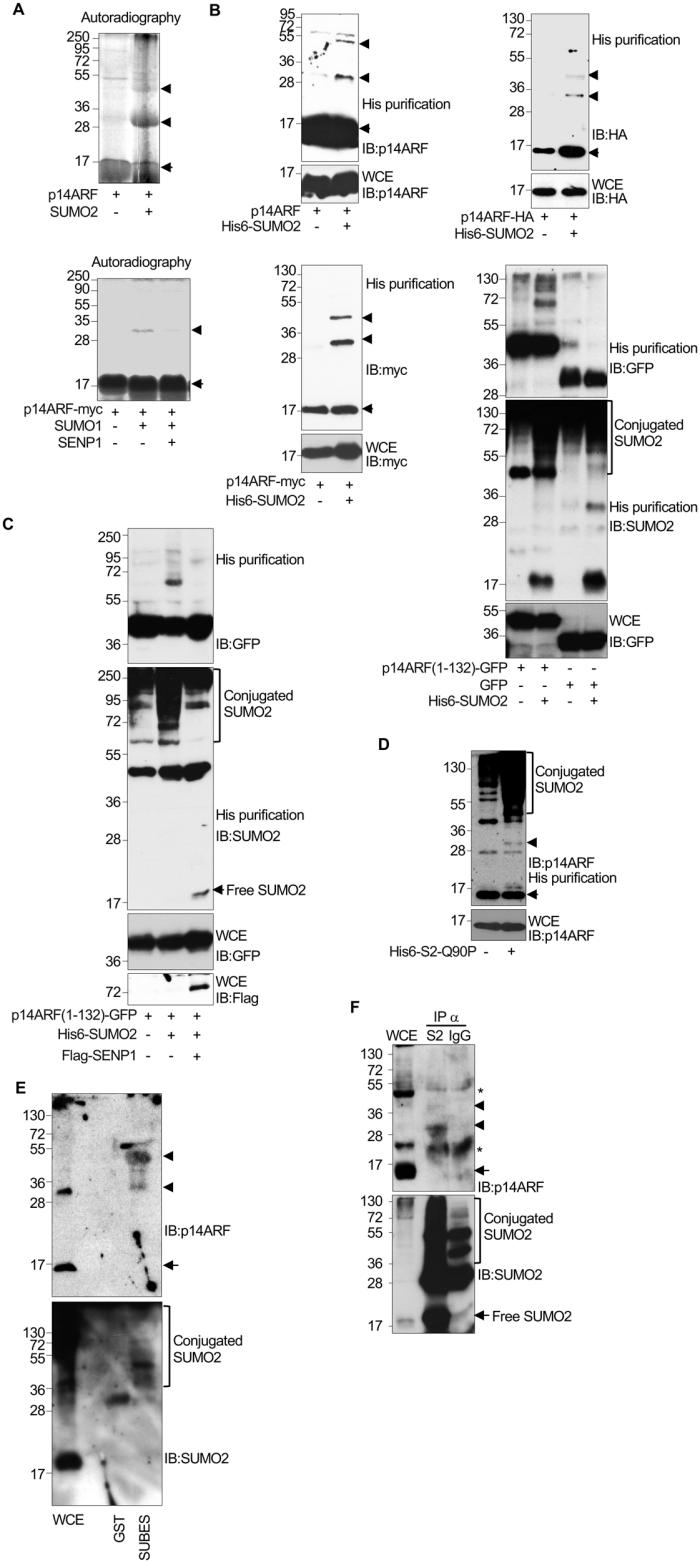
p14ARF is modified by SUMO in vitro and in vivo. **A** In vitro-transcribed/translated ^35^S-methionine-labeled p14ARF protein is modified by SUMO2 in vitro (upper panel). In vitro SUMOylated p14ARF protein was then subjected to an in vitro deSUMOylation assay in the presence of SENP1 (lower panel). **B** Modification of transfected p14ARF (upper left panel), p14ARF-HA (upper right panel), p14ARF-myc (lower left panel), and p14ARF-GFP or GFP (lower right panel) proteins by SUMO2 in HEK-293 cells transfected with His6-SUMO2 and UBC9. **C** Modification of transfected p14ARF-GFP protein by SUMO2 in HEK-293 cells co-transfected with His6-SUMO2, UBC9 and pcDNA or His6-SUMO2, UBC9 and Flag-SENP1. **D** SUMOylation of endogenous p14ARF in PC3 cells transfected with non-deconjugatable His6-SUMO2-Q90P. **E** Detection of endogenously SUMOylated p14ARF using SUBES. **F** Detection of endogenously SUMOylated p14ARF using SUMO2 immunoprecipitation. Arrow indicates unmodified p14ARF protein and both arrowheads and brackets indicate SUMOylated p14ARF. WCE whole cell extracts.

p14ARF interacts with UBC9, the sole SUMO-conjugating enzyme required for SUMOylation [6], and mass spectrometry identified p14ARF as a potential SUMO2 substrate [28], although the precise site of modification was not identified by these authors and our own attempts also failed. To explore whether p14ARF is SUMOylated, we first asked whether SUMO2 can conjugate to in vitro translated ^35^S-methionine-labeled p14ARF protein. SUMOylation causes an increase in apparent molecular weight of around 15 to 20 kDa. The unmodified p14ARF protein was around 15 kDa (Fig. 1A), as expected. Incubation with SUMO2 and the SUMO E1 and E2 enzymes, generated at least two additional higher molecular weight bands: one strong band of around 32 kDa and a fainter one of around 50 kDa molecular weight (Fig. 1A, upper panel), indicating that p14ARF can be modified by SUMO2 in vitro. To confirm this hypothesis, in vitro SUMOylated protein was then subjected to an in vitro deSUMOylation assay. The intensity of the p14ARF-SUMO2 band diminished after incubation with the SUMO specific peptidase SENP1, confirming that it corresponded to SUMOylated p14ARF (Fig. 1A, lower panel).

To evaluate p14ARF SUMOylation in vivo, HEK-293 cells were co-transfected with pcDNA-p14ARF together with pcDNA or His6-SUMO2 and UBC9, and 48 h after transfection, whole protein extracts and histidine-tagged purified proteins were analyzed by western-blot with anti-p14ARF antibody. The unmodified p14ARF protein was detected as a band of around 14 kDa (Fig. 1B, upper left panel), as expected. Although the unmodified protein was detected in all the conditions, likely due to the sticky nature of p14ARF, we observed the appearance of two additional higher molecular weight bands; one strong band of around 32 kDa and a fainter one of around 50 kDa molecular weight (Fig. 1B, upper left panel), only in those cells co-transfected with His6-SUMO2, indicating that p14ARF can be modified by SUMO2 in cells. Similar results were observed after evaluation of the potential SUMO2 modification of p14ARF-HA, p14ARF-Myc, and p14ARF-GFP. Analysis of the His6-SUMO2-conjugated proteins following purification revealed the appearance of bands of the expected p14ARF-HA-SUMO2 and p14ARF-myc-SUMO2 molecular weights in cells co-transfected with His6-SUMO2 (Fig. 1B upper right panel and lower left panel). Bands corresponding with SUMOylated proteins were also detected in the cells co-transfected with His6-SUMO2 and p14ARF-GFP but not in those expressing GFP control (Fig. 1B, lower right panel). In addition, we observed that co-transfection of Flag-SENP1 together with p14ARF-GFP and His6-SUMO2 abolished p14ARF-GFP SUMOylation (Fig. 1C). These results confirmed that p14ARF can be modified by SUMO2 in vivo, independently of the used tag.

To evaluate whether endogenous p14ARF can be SUMOylated, we first used His6-SUMO2-Q90P, a mutant of SUMO2 which cannot be deconjugated from the substrate [29]. PC3 cells (p14ARF WT) were transfected with pcDNA or His6-SUMO2-Q90P and UBC9, and 48 h after transfection whole protein extracts and histidine-tagged purified proteins were analyzed by western-blot with anti-p14ARF antibody. Endogenous p14ARF was detected as a band of ~14 kDa molecular weight (Fig. 1D). In addition, we detected a band of around 32 kDa and additional higher molecular weight bands only in those cells co-transfected with His6-SUMO2-Q90P (Fig. 1D), indicating that SUMO2 modifies endogenous p14ARF.

Finally, we evaluated the SUMOylation of endogenous p14ARF protein in untransfected cells by using SUMO-binding entities (SUBES) [30] or by carrying out an immunoprecipitation assay under denaturing conditions of SUMO2 protein as described previously [31]. Western-blot analysis of p14ARF detected two bands of the expected molecular weights for p14ARF-SUMO protein attached to SUBES but not to the negative control GST (Fig. 1E), indicating that endogenous p14ARF is modified by SUMO2 in cells. Two bands of the expected molecular weights for p14ARF-SUMO2 were also detected in those extracts immunoprecipitated with anti-SUMO2 antibody (Fig. 1F). Collectively, these results demonstrate that p14ARF is a genuine substrate for lysine-independent SUMOylation.

### The N-terminal region of p14ARF is essential for SUMO conjugation

**Fig. 2:**
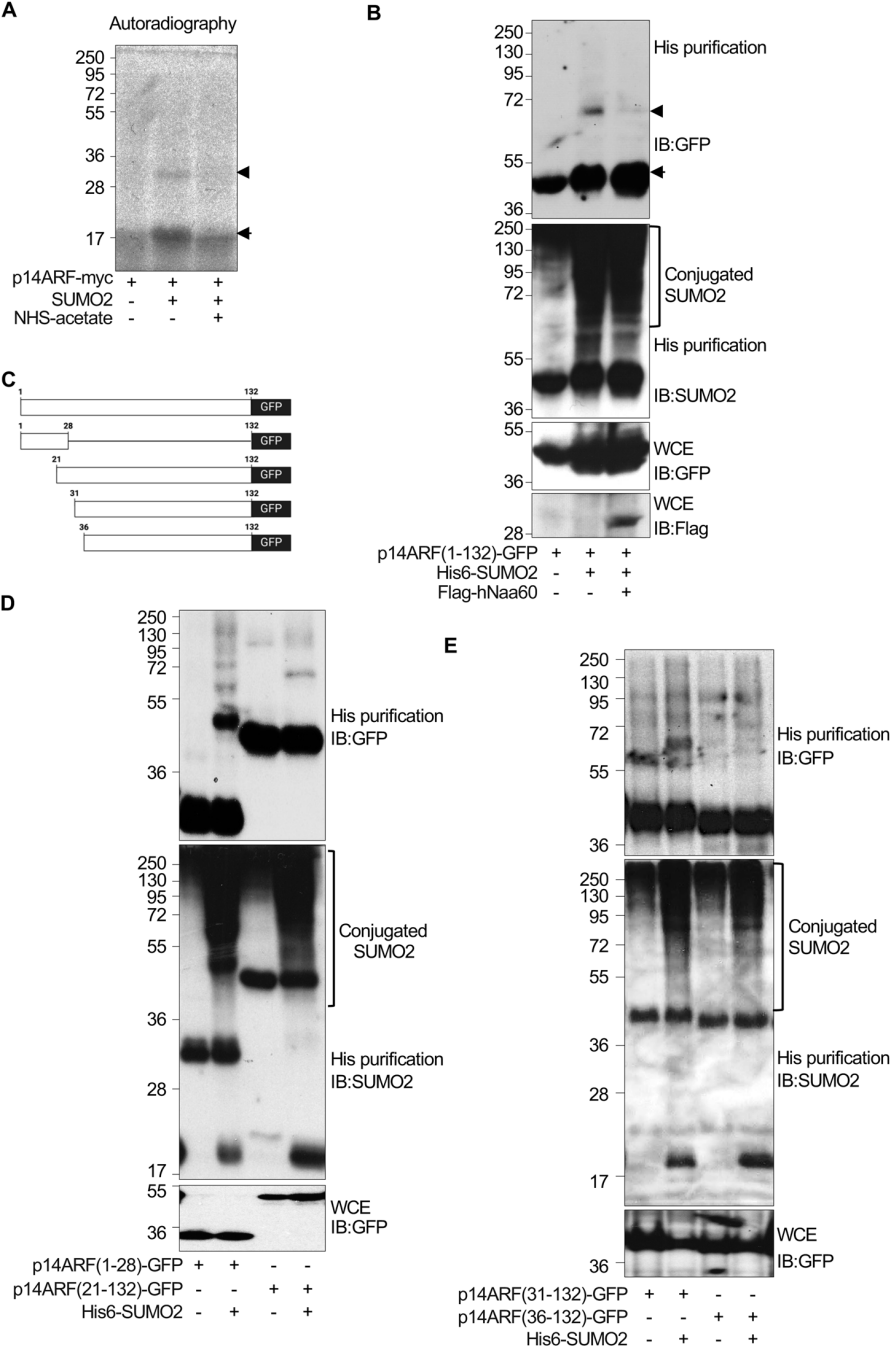
N-terminal domain of p14ARF is essential for SUMO conjugation. **A** In vitro SUMOylation of ^35^S-methionine-labeled p14ARF protein after incubation or not with amino group blocking agent sulfo-NHS-acetate. **B** Modification of transfected p14ARF-GFP protein by SUMO2 in HEK-293 cells co-transfected with His6-SUMO2, UBC9 and pcDNA or His6-SUMO2, UBC9 and Flag-hNaa60. Arrow indicates unmodified p14ARF protein and arrowhead indicates SUMOylated p14ARF. **C** Scheme of constructs encoding C-terminal GFP-tagged deletion mutants of p14ARF. **D** Modification of p14ARF(1–28)-GFP or p14ARF(21-132)-GFP protein by SUMO2 in HEK-293 cells transfected with His6-SUMO2 and UBC9. **E** Modification of p14ARF(31-132)-GFP or p14ARF(36-132)-GFP protein by SUMO2 in HEK-293 cells transfected with His6-SUMO2 and UBC9. WCE whole cell extracts.

Although p14ARF protein is a lysine-less protein, it can be modified by ubiquitin at the N-terminal-NH2 group [19, 20]. Therefore, we decided to analyze the role of the N-terminus in SUMOylation. ^35^S-methionine in vitro translated p14ARF protein was treated or not with 1 mM sulfo-NHS-acetate for 1 h at room temperature to block amine groups. We then carried out an in vitro SUMOylation assay with SUMO2 and using sulfo-NHS-acetate-treated or untreated p14ARF protein as a substrate. Incubation of untreated p14ARF with SUMO2 generated a band corresponding to the expected p14ARF-SUMO2 molecular weight (Fig. 2A). A p14ARF-SUMO2 band was not observed in the lane corresponding to sulfo-NHS-acetate-treated protein (Fig. 2A), suggesting that SUMO2 likely conjugates to the N-terminal-NH2 group of p14ARF.

To further evaluate the role of the N-terminal amino group on p14ARF SUMOylation, based on the strategy developed by Weng et al. [27], we evaluated the effect of expressing the N-alpha acetyltransferase 60 (Naa60) that specifically mediates the acetylation of a substrate at the N-terminus in an irreversible manner, on p14ARF SUMOylation. HEK-293 cells were co-transfected with plasmids encoding for p14ARF-GFP together with pcDNA3, UBC9 and His6-SUMO2, or UBC9, His6-SUMO2 and Flag-hNaa60 and 48 h after transfection, whole cell protein extracts and histidine-tagged purified proteins were analyzed by western-blot with anti-GFP or anti-SUMO2 antibody. Co-expression of hNaa60 abolished p14ARF-GFP SUMOylation (Fig. 2B), indicating that the N-terminal amino group of p14ARF is essential for its SUMOylation.

Finally, we analyzed the SUMOylation of a series of p14ARF-GFP deletion constructs (Fig. 2C). HEK-293 cells were co-transfected with plasmids encoding for p14ARF-GFP deletion constructs together with pcDNA3 or UBC9 and His6-SUMO2, and 48 h after transfection, whole cell protein extracts and His6-SUMO2-conjugated proteins were analyzed by western-blot with anti-GFP or anti-SUMO2 antibody. Bands corresponding to SUMOylated ARF protein were observed after co-expressing His6-SUMO2 and a construct consisting of the first 28 amino acid residues of p14ARF (Fig. 2D), a p14ARFconstruct with deletion of the first 20 residues (Fig. 2D), or a p14ARF protein with deletion of the first 30 residues (Fig. 2E). However, deletion of the first 35 residues of p14ARF abolished its SUMOylation (Fig. 2E). All together, these data confirmed that the N-terminal region of p14ARF is essential for p14ARF SUMOylation.

### SUMO promotes p14ARF protein stability

**Fig. 3:**
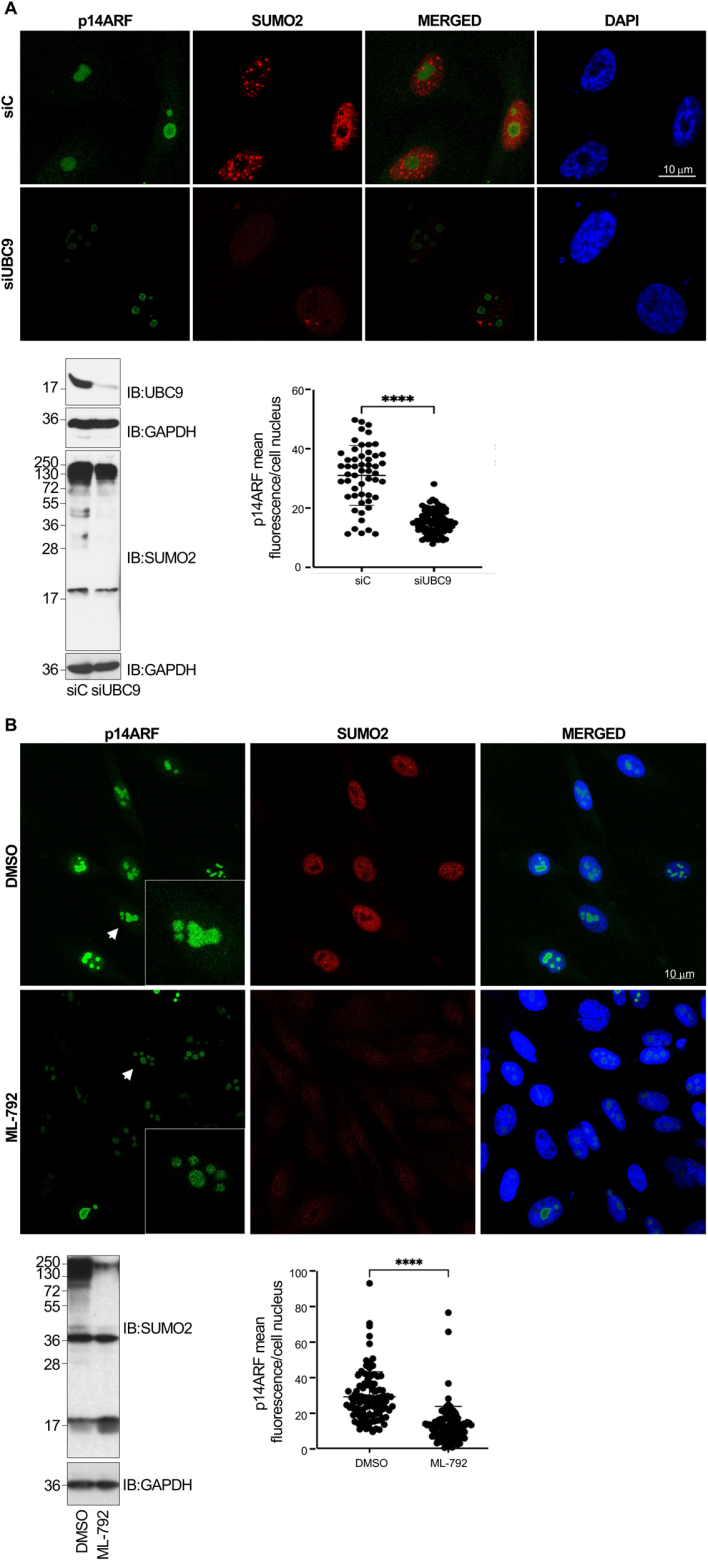
Inhibiting SUMOylation reduces the intensity of p14ARF signal. **A** Representative confocal images of PC3 cells transfected with siRNA control (siC) or siRNA against UBC9 (siUBC9), and immunostained using anti-p14ARF and anti-SUMO2 antibodies (upper panel). Quantification of p14ARF fluorescence intensity for cells transfected with siC or siUBC9 are shown on the lower right panel. Western-blot analysis of PC3 cells transfected with siRNA against UBC9 (siUbc9) or siRNA control (siC) is shown in lower left panel. **B** Representative confocal images of PC3 cells 16 h after treatment with DMSO or the SUMOylation inhibitor ML-792, and immunostained using anti-p14ARF and anti-SUMO2 antibodies. Quantification of p14ARF fluorescence intensity for cells treated with DMSO or ML-792 are shown on the lower right panel. Mean nuclear fluorescence intensity between siC and siUBC9 transfected cells or between DSMSO and ML-792 treated cells were compared. Western-blot analysis of PC3 cells treated with DMSO or ML-792 is shown in lower left panel. Each dot represents a nucleus and error bars the standard deviation (SD); *n* = 55–100 from at least two independent biological replicates. ****P < 0.0001, Student’s *t*-test.

SUMO conjugation can modulate protein stability, subcellular localization, or activity [32]. To evaluate the impact of SUMOylation on p14ARF, we first carried out an immunofluorescence assay with anti-p14ARF and anti-SUMO2 antibodies on PC3 cells transfected with siRNA for UBC9 to downmodulate SUMOylation, as shown in Fig. 3A lower left panel and Supplementary Fig. S1. p14ARF protein was nucleolar in cells transfected with siRNA control (siC), as expected (Fig. 3A, upper panel) [33, 34]. SUMO2 was mainly detected in the nucleus and some co-localization with p14ARF protein within the nucleolus could be observed in some cells (Fig. 3A, upper panel). Transfection with siRNA against UBC9 (siUBC9) did not alter the subcellular localization of p14ARF but significantly reduced p14ARF signal (Fig. 3A, upper and lower right panels), suggesting that SUMOylation increases the steady-state levels of p14ARF. To further test this possibility, we carried out immunofluorescence assays with anti-p14ARF and anti-SUMO2 antibodies on PC3 cells treated with ML-792 (inhibitor of the SUMO E1-enzyme) in order to inhibit SUMOylation (Fig. 3B, lower left panel and Supplementary Fig. S2) or DMSO as a control. Indeed, ML-792 treatment significantly reduced p14ARF signal without altering its subcellular localization, supporting our hypothesis (Fig. 3B, upper and lower right panels).

**Fig. 4:**
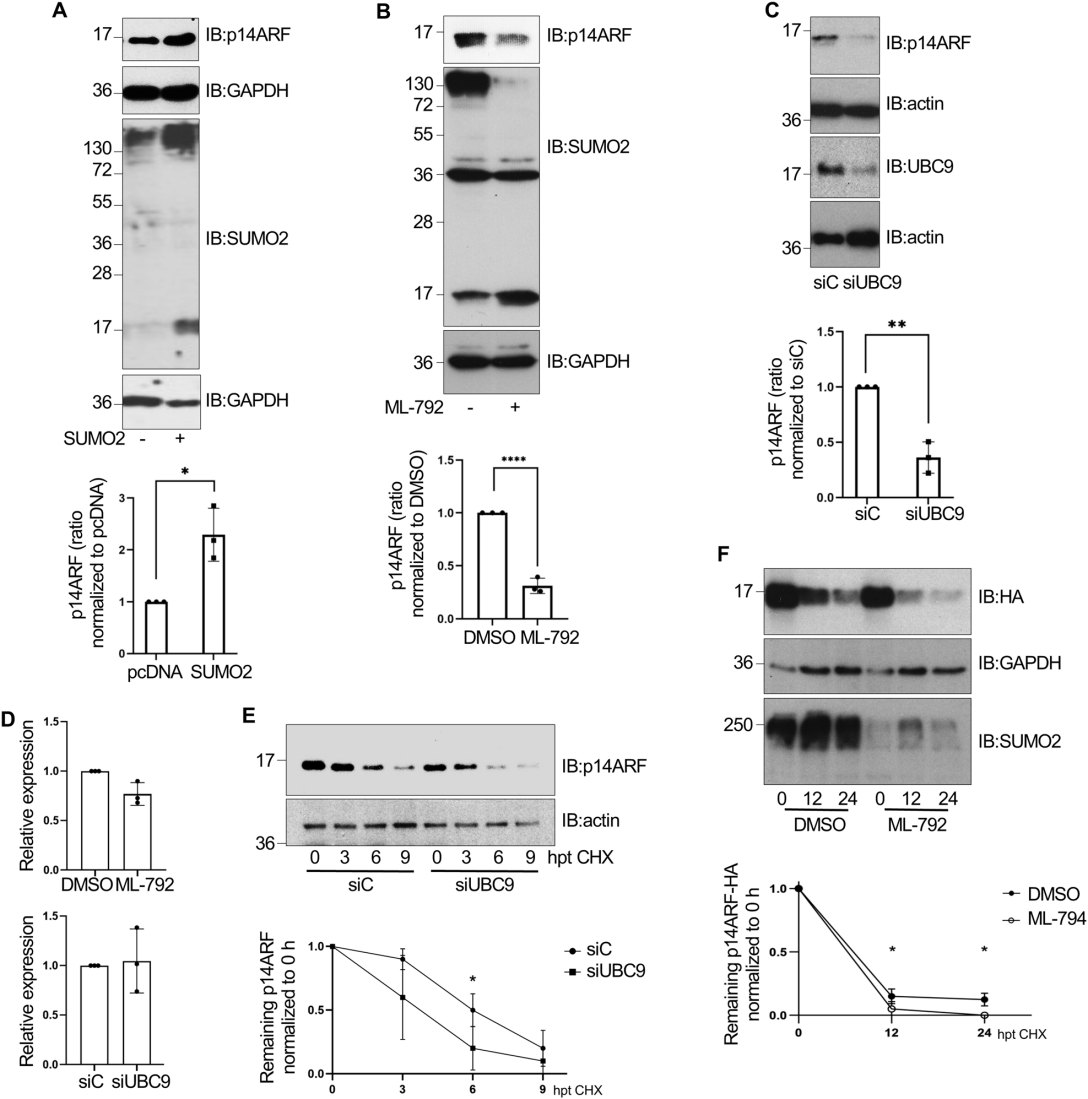
SUMOylation stabilizes p14ARF. **A** PC3 cells were transfected with SUMO2 or the empty vector pcDNA. At 48 h after transfection, p14ARF levels were analyzed by western-blot (upper panel). **B** PC3 cells were treated with the SUMOylation inhibitor ML-792 or DMSO and 16 h after treatment, the levels of p14ARF were analyzed by western-blot (upper panel). **C** PC3 cells were transfected with siRNA against UBC9 (siUBC9) or siRNA control (siC). At 72 h after transfection, UBC9 and p14ARF protein levels were analyzed by western-blot (upper panel). The intensity of the bands in (**A**–**C**) was quantified using ImageJ software. Intensity of p14ARF bands were normalized to GAPDH or actin bands and plotted (lower panels). Columns are representative of the mean and error bars represent the standard deviation of three biological replicas. Statistical analysis was assessed by a Student’s *t*-test. *P < 0.05; **P < 0.005; ****P < 0.0001. **D** PC3 cells were treated as indicated in (**B**) or transfected as indicated in (**C**). RNA was extracted and expression of mRNAs encoding p14ARF was analyzed by QRT-PCR. Values were normalized to GAPDH expression. Columns are representative of the mean and error bars represent the standard deviation of three biological replicas. **E** PC3 cells were transfected with siUBC9 or siC and 48 h after transfection (hpt), cells were treated with cycloheximide (CHX) and, at different times after treatment, levels of p14ARF were evaluated by western-blot analysis using anti-p14ARF antibody. p14ARF bands intensity were normalized to actin bands for each respective time and plotted (lower panel). Data are representative of the mean and error bars represent the standard deviation of three biological replicas. Statistical analysis was assessed by a Student’s *t*-test. *P < 0.05. **F** HEK-293 cells were transfected with p14ARF-HA and 48 h after transfection, cells were treated with DMSO or ML-792 for 4 h. Then, cells were treated with cycloheximide (CHX) in the presence or absence of ML-792 and, at different times after treatment, levels of p14ARF-HA protein were evaluated by western-blot with anti-HA antibody. p14ARF-HA bands were normalized to GAPDH bands for each respective time and plotted (lower panel). Data are representative of the mean and error bars of three biological replicas. Statistical analysis was assessed by a Student’s *t*-test. *P < 0.05.

The impact of SUMOylation on p14ARF protein levels was then evaluated by western-blot analysis. Transfection of PC3 cells with SUMO2 increased p14ARF protein levels (Fig. 4A). In contrast, treatment with the SUMOylation inhibitor ML-792 or transfection with siUBC9 reduced p14ARF protein levels (Fig. 4B, C). We did not observe significant differences in p14ARF mRNA levels between cells treated with ML-792 and DMSO or between cells transfected with siC and siUBC9 (Fig. 4D), suggesting that SUMO may stabilize p14ARF protein. To further define the role of SUMO on p14ARF stability, we investigated the effects of downmodulating UBC9 on p14ARF half-life, using cycloheximide (CHX) to block de novo protein synthesis in PC3 cells. Transfection of siUBC9 reduced the stability of endogenous p14ARF protein (Fig. 4E). Similarly, treatment with the SUMOylation inhibitor ML-792 reduced the stability of transfected p14ARF-HA protein (Fig. 4F). All together, these results indicated that SUMOylation promotes p14ARF stability.

### Blocking ubiquitination with TAK-243 or MLN4924 promotes p14ARF SUMOylation

**Fig. 5:**
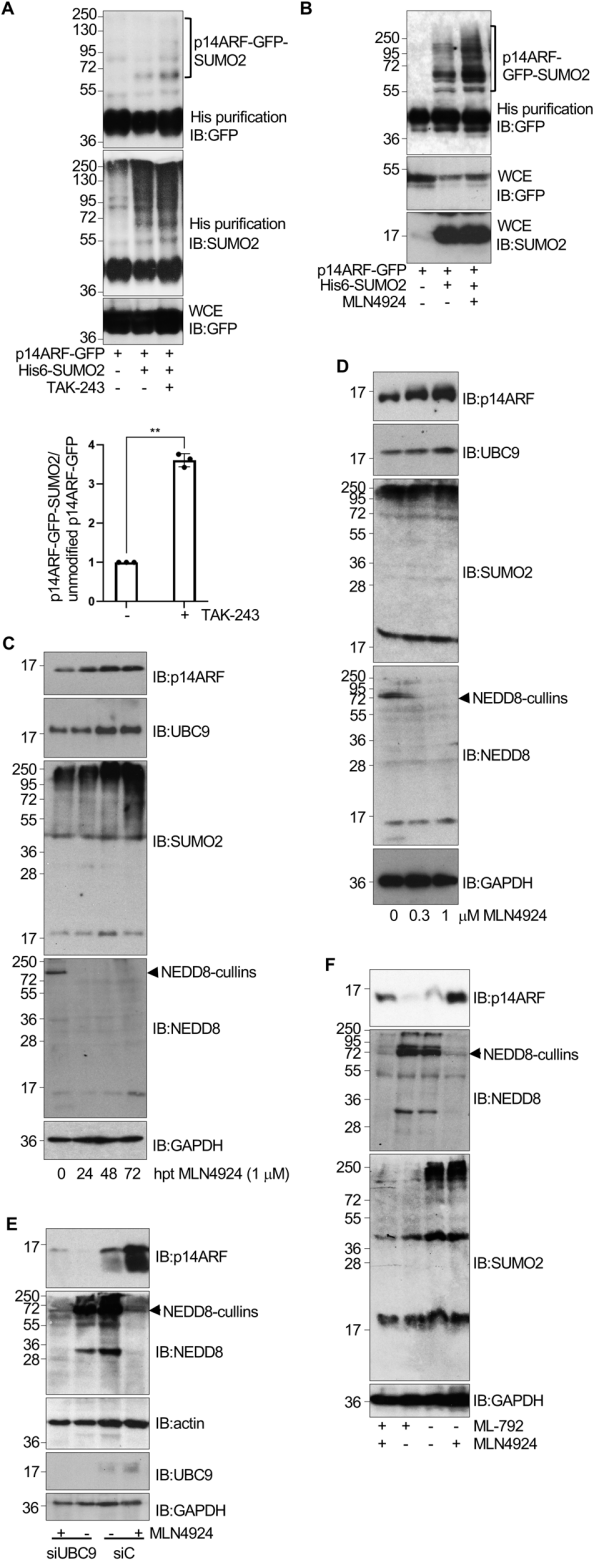
Blocking ubiquitylation with TAK-243 or MLN4924 promotes p14ARF SUMOylation. Modification of transfected p14ARF-GFP by SUMO2 in HEK-293 cells transfected with His6-SUMO2 and UBC9 and treated or not with TAK-243 (**A**) or MLN4924 (**B**) for 24 h. WCE, whole cell extracts. The intensity of SUMO-conjugated and unmodified bands in (**A**) was quantified using ImageJ software, and the ratio of SUMO2-modified to unmodified p14ARF-GFP protein was calculated (lower panel). Columns are representative of the mean and error bars represent the standard deviation of three biological replicas. Statistical analysis was assessed by a Student’s *t*-test. **P < 0.005. **C** PC3 cells were treated with MLN4924 (1 μM) and at different times after treatment, protein extracts were analyzed by western-blot with the indicated antibodies. hpt hours post-treatment. **D** PC3 cells were treated with the indicated concentrations of MLN4924 and 48 h after treatment protein extracts were evaluated by western-blot analysis with the indicated antibodies. **E** PC3 cells were transfected with siRNA against UBC9 (siUBC9) or siRNA control (siC) and 48 h after transfection, cells were treated with DMSO or MLN4924. At 48 h after treatment protein extracts were analyzed by western-blot with the indicated antibodies. **F** PC3 cells were treated with DMSO, ML-792, MLN4924 or a combination of both ML-792 and MLN4924 and, 48 h after treatment, protein extracts were analyzed by western-blot with the indicated antibodies.

One of the mechanisms that governs degradation of p14ARF is N-terminal ubiquitin conjugation [19, 20], which can be mediated by Cullin-RING E3 ligases [21, 22]. A hypothesis is that SUMO and ubiquitin compete for the p14ARF N-terminus, causing stabilization or destabilization, respectively. If ubiquitination and SUMOylation compete then blocking the former should induced p14ARF SUMOylation and stability. To test this idea, we employed two different strategies to inhibit p14ARF ubiquitination. First, we tested whether TAK-243, which inhibits the ubiquitin activation enzyme (E1) [35] induced p14ARF SUMOylation. HEK-293 cells were co-transfected with p14ARF-GFP together with pcDNA3 or UBC9 and His6-SUMO2 and then cells were treated with DMSO or TAK-243 for 24 h. Analysis of the histidine-tagged purified proteins revealed the presence of SUMOylated p14ARF in the cells transfected with His-SUMO2 and treated with DMSO, as expected (Fig. 5A). A significant increase in the levels of SUMOylated p14ARF was observed after TAK-243 treatment (Fig. 5A), indicating that inhibiting ubiquitination facilitates the conjugation of SUMO2 to p14ARF. Next, we tested MLN4924, which inhibits Cullin NEDDylation thus preventing CRL-mediated ubiquitination of their targets, including p14ARF [21, 22]. A clear increase in the levels of SUMOylated p14ARF was observed at 24 h after MLN4924 treatment (Fig. 5B), indicating that inhibition of NEDDylation also facilitates the conjugation of SUMO2 to p14ARF.

Our data raises the possibility that SUMOylation may underpin the MLN4924-induced upregulation of p14ARF. First, we confirmed a time and dose-dependent increase in p14ARF levels in in PC3 prostate cancer cells exposed to MLN4924 (Fig. 5C, D). An increase in UBC9 and SUMO2 levels was also observed after 48 h of MLN4924 treatment (Fig. 5C, D). Based on these observations, we predicted that the accumulation of p14ARF resulting from inhibiting NEDDylation should be dependent on SUMOylation. To test this idea, PC3 cells were transfected with siC or siUBC9 and at 48 h after transfection, cells were treated or not with the NEDDylation inhibitor MLN4924 for an additional 48 h period, after which we evaluated p14ARF levels. Depleting UBC9 strongly reduced the levels of p14ARF protein, as before (Fig. 5E). MLN4924 treatment rescued the siUBC9-induced degradation of p14ARF although not quite back to levels in siC treated control cells (Fig. 5E). Similar results were also observed in H1299 cells (Supplementary Fig. S3). We also evaluated the impact on p14ARF levels of co-treating the cells with the NEDDylation inhibitor MLN4924 and the SUMOylation inhibitor ML-792. Indeed, the decrease of p14ARF protein in ML-792 treated PC3 cells was rescued by MLN4924 to levels above untreated controls but below cells treated with MLN4924 alone (Fig. 5F). All together these data support a model in which p14ARF SUMOylation counteracts ubiquitination and degradation to stabilize p14ARF.

### p14ARF-dependent induction of SUMOylation

**Fig. 6:**
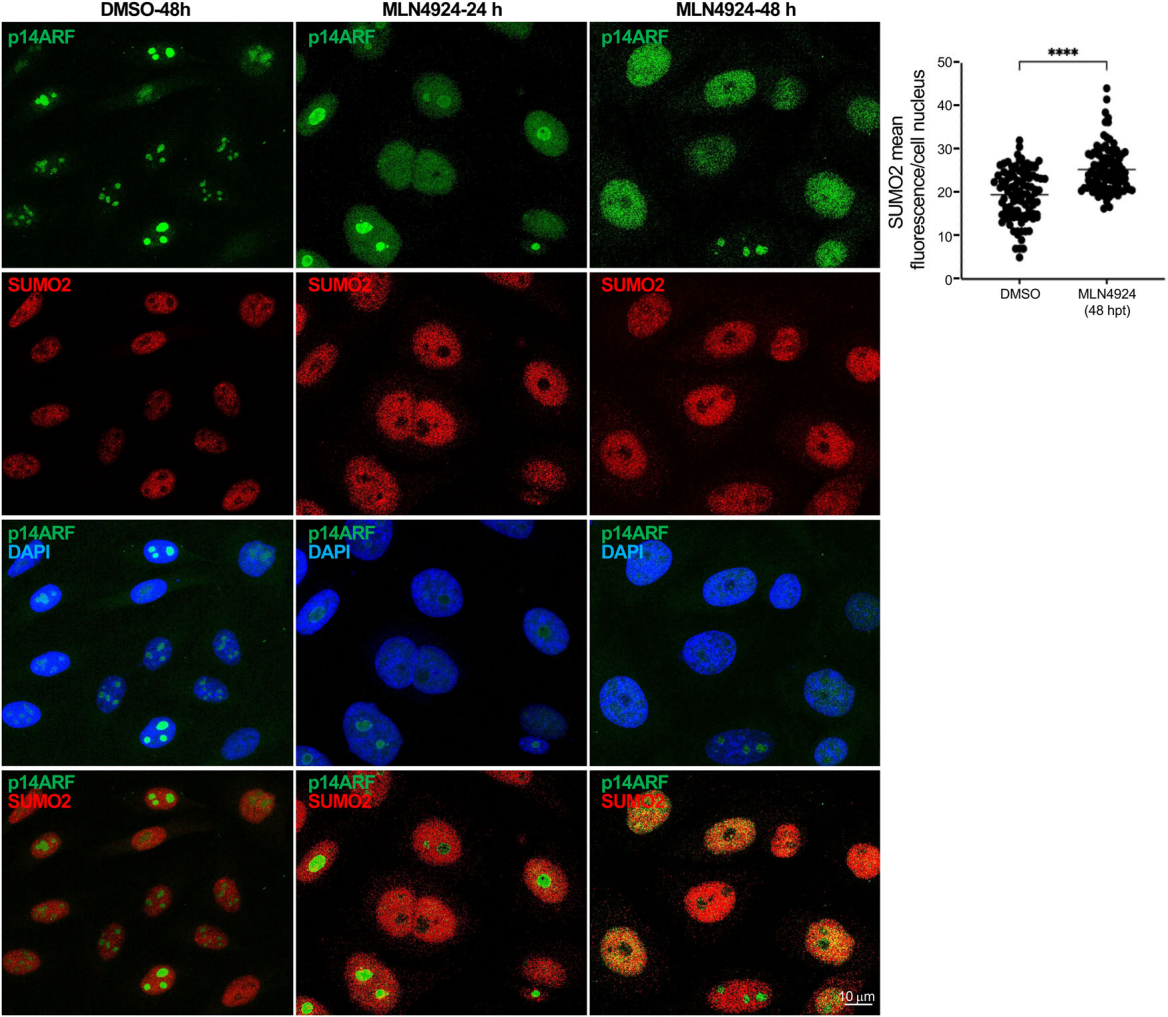
Effect of the NEDDylation inhibitor MLN4924 on p14ARF and SUMO2 by immunofluorescence. PC3 cells were treated with MLN4924 and 24 or 48 h post-treatment (hpt), cells were immunostained with anti-p14ARF and anti-SUMO2 antibodies (left panel). Quantification of SUMO2 fluorescence intensity for cells treated with DMSO or MLN4924 during 48 h are shown on the right. Mean nuclear fluorescence intensity between DMSO and MLN4924 treated cells were compared. Each dot represents a nucleus and error bars the SD; *n* = 100. ****P < 0.0001, Student’s *t*-test.

**Fig. 7:**
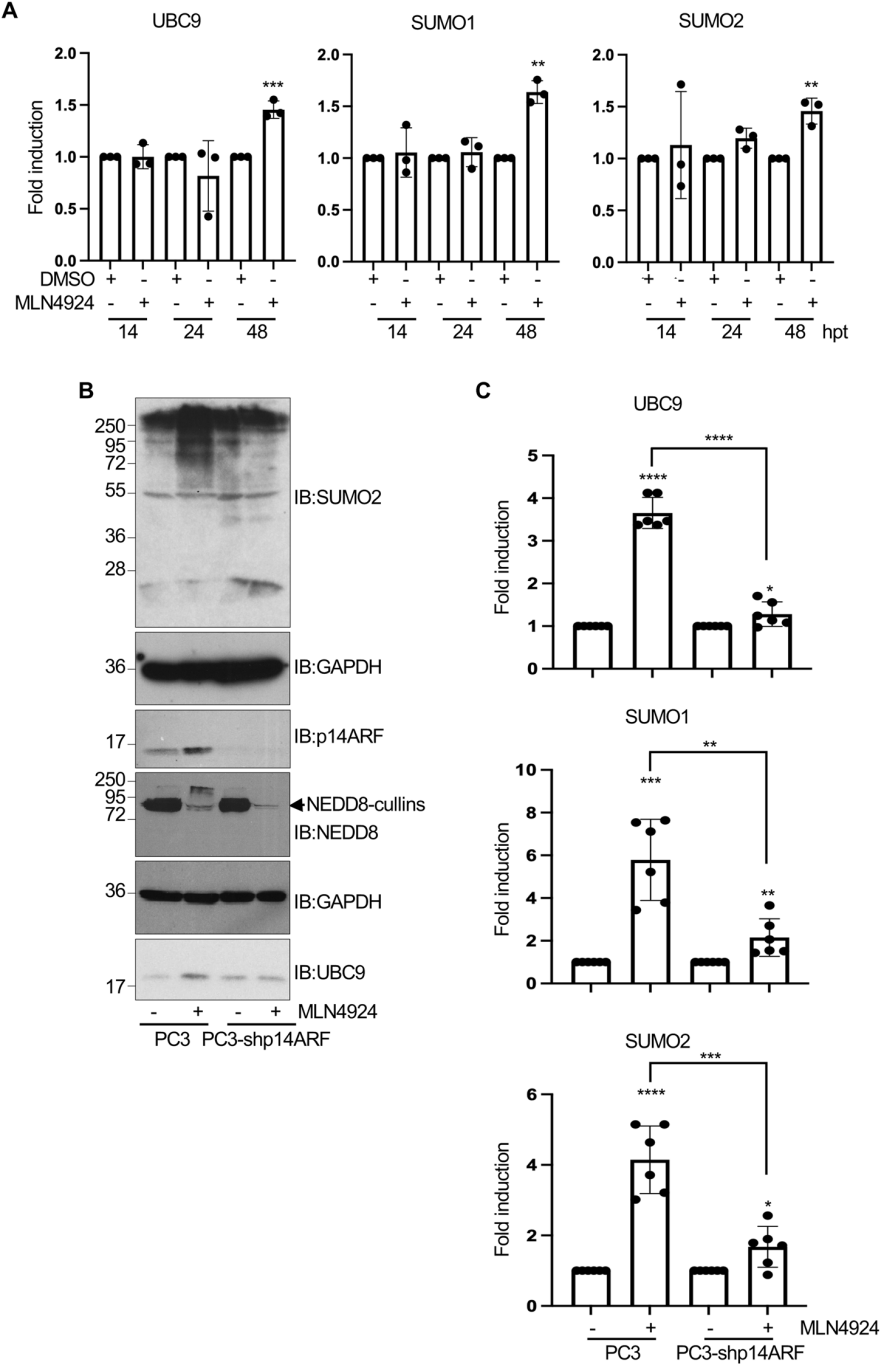
The upregulation of SUMOylation in response to MLN4924 treatment requires p14ARF. **A** PC3 cells were treated with DMSO or MLN4924 and, at different times after treatment, UBC9, SUMO1, and SUMO2 were analyzed by QRT-PCR. Values were normalized to GAPDH expression. Columns are representative of the mean and error bars represent the SD of three biological replicas. Statistical analysis was assessed by Student’s *t*-test. **P < 0.005; ***P < 0.0005. **B** PC3 or PC3-shp14ARF cells were treated with MLN4924 or DMSO and 48 h after treatment, protein extracts were analyzed by western-blot with the indicated antibodies. **C** PC3 or PC3-shp14ARF cells were treated with DMSO or MLN4924 and 48 h after treatment, transcription of UBC9, SUMO1, and SUMO2 were analyzed by QRT-PCR. Values were normalized to GAPDH expression. Columns are representative of the mean and error bars represent the SD of six biological replicas. Statistical analysis was assessed by Student’s *t*-test. *P < 0.05; **P < 0.005; ***P < 0.0005; ****P < 0.0001. hpt hours post-treatment.

We have shown that MLN4924 increases p14ARF, UBC9 and SUMO2 protein levels as well as the conjugation of SUMO2 to p14ARF. An increase in SUMO2 levels in PC3 cells upon treatment with MLN4924 was also detected by immunofluorescence analysis (Fig. 6). This increase in SUMO2 signal correlated with a shift in p14ARF localization from the nucleolus to the nucleoplasm (Fig. 6), consistent with the effect of MLN4924 on some other nucleolar proteins [4, 36]. RT-qPCR analyses revealed a modest ~1.5-fold increase in UBC9, SUMO1 and SUMO2 mRNAs 48 h after MLN4924 exposure, indicating that the observed increase in the levels of SUMOylation components upon NEDDylation inhibition is due, at least partially, to transcriptional regulation (Fig. 7A). Strikingly, short hairpin RNA knockdown of p14ARF (shp14ARF) in PC3 cells revealed that the ability of MLN4924 to augment UBC9 protein and SUMO2-conjugated protein levels requires p14ARF (Fig. 7B). Moreover, MLN4924-driven induction of UBC9, SUMO1 and SUMO2 mRNAs was also affected by p14ARF deficiency (Fig. 7C). Thus, p14ARF contributes to the upregulation in SUMOylation induced by MLN4924 at transcriptional and post-transcriptional level for critical SUMO components.

### Tumor suppressor p14ARF contributes to the cytotoxic activity of MLN4924

**Fig. 8:**
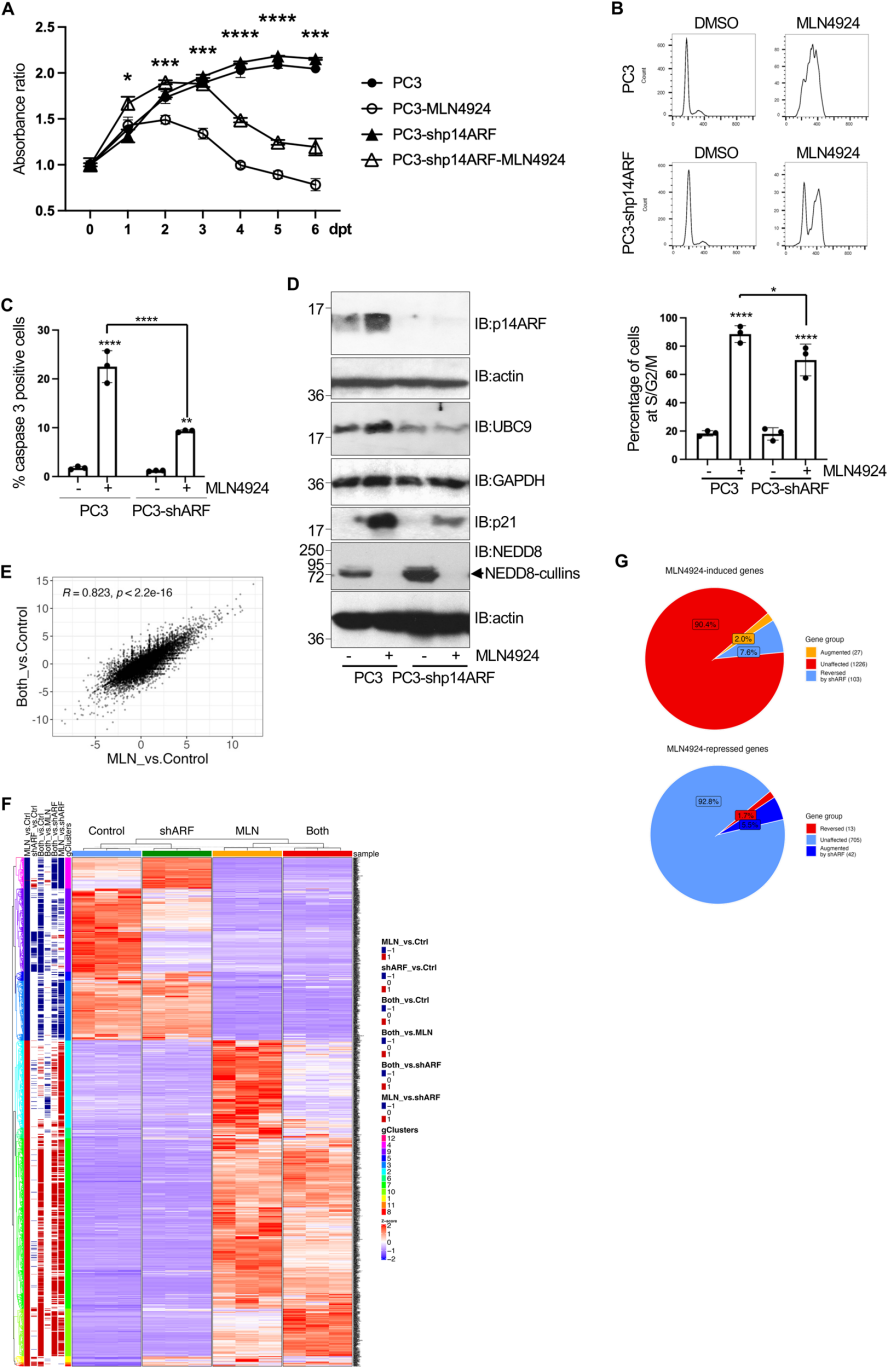
Impact of p14ARF on the cytotoxic effect of MLN4924 on PC3 cells. **A** MTT of PC3 cells or p14ARF-silenced PC3 (PC3-shp14ARF) cells at different times after MLN4924 treatment. Data are representative of the mean and error bars represent the SD of five samples. Statistical analysis of the significant differences between PC3 and PC3-shARF cells, treated with MLN4924 was assessed by a Student’s *t*-test. *P < 005; ***P < 0.0005; ****P < 0.0001. **B** Cell cycle analysis of PC3 or PC3-shARF at 48 h after treatment with DMSO or MLN4924. The percentage of cells at S/G2/M in each condition was plotted in histograms. Results are the mean of three biological replicas. Statistical analysis was assessed by ANOVA one way. *P < 0.05; ****P < 0.0001. **C** PC3 or PC3-shp14ARF cells were treated with DMSO or MLN4924 and at 5 days after treatment, cells were stained using anti-caspase 3 antibody and then cells were analyzed by cytometry. Columns are representative of the mean and error bars represent the SD of three biological replicas. Statistical analysis was assessed by ANOVA one way. **P < 0.005; ****P < 0.0001. **D** Western-blot analysis of PC3 or PC3-shp14ARF cells at 48 h upon treatment with MLN4924 with the indicated antibodies. **E** Scatter plot of the log2 fold changes in the “Both vs Ctrl” and “MLN vs Ctrl” RNA-seq assays. **F** Induced or repressed MLN4924 targets (*p* < 0.05, log2FC > 2 < −2) are displayed as *Z*-scores across all four conditions (PC3 cells or PC3-shp14ARF cells at 72 h after DMSO or MLN4924 treatment) with hierarchical clustering on both genes (rows) and samples (columns). The annotation column “gClusters” indicates gene clusters, and the other six annotation columns on the left indicate the significantly induced (red) or repressed (blue) genes in the indicated comparison e.g., the “MLN vs Ctrl” group indicates genes altered by MLN4924, while the “Both vs MLN” group indicates which MLN4924-altered genes are altered by shARF; genes with opposite colors in these two columns indicate cases where shARF reversed the effect of MLN4924. Full gene lists are provided in Supplementary Table 1. **G** Pie charts showing the percentage of MLN4924-induced or repressed genes (i.e., the DEGs shown in **F**) that are affected by ARF depletion (i.e., also *p* < 0.05 and log2FC > 2 < −2 in Both vs MLN comparison).

Our work and prior studies show that MLN4924 promotes p14ARF stability [21, 22], but the extent to which p14ARF is critical for drug efficacy is unknown. Inhibiting CRLs with MLN4924 stabilizes multiple tumor suppressors (e.g., p53, p21, p27, WEE1, IKBA, CDT1 etc.) triggering growth arrest, DNA damage and apoptosis of prostate cancer, including PC3 cells [37–40]. MLN4924 also blocks NEDDylation of proteins other than Cullins, such as ribosomal proteins [41]. In view of the pleiotropic effects of MLN4924, the relative importance of any one effector, such as p14ARF, is difficult to predict. Our study also reveals that MLN4924 induces SUMO and Ubc9 in a p14ARF-dependent manner (Fig. 7), but as the drug likely affects hundreds of genes, whether other MLN4924-altered genes exhibit such dependency is unclear. Thus, we next assessed whether and to what extent p14ARF contributes to MLN4924-induced cytotoxicity and transcriptome modulation. To evaluate whether p14ARF is an MLN4924 effector we first carried out an MTT (3-(4,5-Dimethylthiazol-2-yl)-2,5-Diphenyltetrazolium Bromide) assay on PC3 or PC3-shp14ARF cells at different days after treatment with MLN4924 or DMSO. We did not observe significant differences between the proliferation of PC3 and PC3-shp14ARF cells grown in the presence of DMSO (Fig. 8A). Treatment with MLN4924 reduced the number of viable PC3 cells, as previously reported [37, 39, 42], but p14ARF depletion increased viability, albeit modestly (Fig. 8A). Consistent with these data, depleting p14ARF reduced somewhat the ability of MLN4924 both to arrest PC3 cells in G2/M and induce apoptosis (Fig. 8B, C). Thus, p14ARF contributes partially to MLN4924 efficacy.

The above results suggest that p14ARF depletion should also partially alter the molecular effects of MLN4924. Multiple studies have shown that the CDK inhibitor p21 contributes to drug efficacy [36, 43–46]. In PC3 cells MLN4924-induced p21 protein considerably, which was partially reduced when p14ARF was depleted, consistent with the partial rescue of drug cytotoxicity (Fig. 8D).

Next, we investigated to what extent depleting p14ARF reverses the transcriptional effects of MLN4924. RNA-seq was performed on RNA from control or p14ARF-depleted PC3 cells treated with DMSO or MLN4924 (“MLN”) for 3 days. Six possible pairwise comparisons exist: *i*. MLN vs Ctrl, *ii*. shARF vs Ctrl, *iii*. Both vs Ctrl, *iv*. Both vs MLN, *v*. Both vs shARF, *vi*. MLN vs shARF. Lists of DEGS and GSEA data are provided (Supplementary Table 1 sheets F–M) but none of these pairwise comparisons answer the main question. For example, while Both vs MLN reveals MLN targets that shARF increases or decreases, it does not reveal whether the MLN targets were induced or repressed by MLN. Rather, it is necessary to extract the relevant genes from two pairwise comparisons: “MLN vs Ctrl” reveals genes that MLN activates and represses, then the subset of that list that is affected in the “Both vs MLN” comparison reveals the four types of shARF affected MLN targets: MLN-induced genes that shARF represses or further activates, and MLN-repressed genes that shARF activates or further represses. A scatter plot comparing the fold-change of differentially regulated genes (DEGs) in “MLN vs control” compared to “Both vs control” revealed a highly significant correlation (Fig. 8E), suggesting that ARF depletion does not substantially alter the effects of MLN4924 3 days after drug treatment. From the “MLN vs Ctrl” comparison, the drug induced 1356 and repressed 760 genes (*p* < 0.05, log2FC > 2 < −2). Overlaying those targets with DEGs from the “Both vs MLN” comparison revealed that consistent with the correlation data (Fig. 8E), p14ARF depletion reversed only 103 (7.6%) and augmented only 27 (2.0%) MLN4924-induced genes, and it reversed only 13 (1.7%) and augmented only 42 (5.5%) of MLN4924-repressed genes (cutoff at *p* < 0.05, log2FC > 2 < −2; Fig. 8F, G, Supplementary Table 1 sheets B, C). Among MLN4924-induced/repressed genes that were reversed by shARF, gProfiler analysis found no or few Kegg pathways enriched for each of the four gene sets, and even for significant pathways the *p* values and number of genes (relative to the number in the entire gene set) were modest (Supplementary Table 1, sheet B). Thus, depleting p14ARF has modest effects on either MLN-induced or MLN-enriched targets. Lowering the stringency for the “Both vs MLN” comparison to log2FC > 1 or <−1 increased the number of MLN4924 targets that shARF reversed, but did not markedly increase the discovery of enriched Kegg pathways (Supplementary Table 1, sheets D, E). Thus, depleting p14ARF has a minor effect on the MLN4924 disrupted transcriptome. As we only measured the transcriptome 3 days after drug treatment, it is unclear whether p14ARF depletion may affect more of the early gene expression changes caused by MLN4924; for example, the p14ARF-dependent induction of UBC9 and SUMO seen at 48 h (Fig. 7C) was not observed 72 h after drug treatment (Supplementary Table 1). These mild transcriptional effects suggest that the partial rescue of MLN4924 treated cells by ARF depletion may primarily reflect post-transcriptional effects on proteins such as p21 (Fig. 8D).

## Materials and methods

### Cell lines and reagents

Commercial human embryonic kidney 293 (HEK-293) (CRL-1573) and the prostate cancer cell line PC3 (ATCC)(CRL-1435) were cultured in complete medium (DMEM, supplemented with 10% fetal bovine serum, 1% penicillin/ streptomycin). Cell lines were routinely confirmed to be mycoplasma-free. PC3 cells were authenticated by STR Profiling (SECUGEN) within the last 2 months. The cells were transfected using Xtreme-Gene siRNA (Roche, Barcelona, Spain) or PEI (Polysciences, Hirschberg an der Bergstrasse, Germany) transfection reagents, following manufacturers’ instructions. Cycloheximide (CHX) and NEDDylation inhibitor MLN4924 were from Millipore Sigma. SUMOylation inhibitor (ML-792) was purchased from Quimigen. Smart-pool small interfering RNAs against UBC9 (siUBC9) and scramble small interfering RNA (siC) were purchased from Dharmacon (Madrid, Spain). Stably transfected cells were generated by transducing PC3 cells with retrovirus supernatant obtained from HEK-293 cells co-transfected with retrovirus vector and shp14ARF. Seventy-two hours after transduction, the PC3 cells were selected by growing in medium supplemented with Hygromycin B (500 µg/ml) for 2 weeks. MLN4924 was used at 1 μM unless otherwise indicated. ML-792 was used at 500 nM.

### Plasmids and antibodies

p14ARF 3XHA (p14ARF-HA) was a gift from Jaewhan Song (Addgene plasmid 78764) [47]. pcDNA3-p14ARF was kindly provided by Susana Llanos [16]. pcDNA-histidine 6 (His6)-SUMO1, pcDNA-His6-SUMO2, and pcDNA-SV5-UBC9 plasmids were previously described [48, 49]. The GFP-tagged ARF constructs were previously reported [50]. shRNA plasmid against human p14ARF was kindly provided by Dr. R Agami (Netherlands Cancer Institute, Netherlands) [51]. Flag-SENP1 was a gift from Edward Yeh (Addgene plasmid # 17357; http://n2t.net/addgene:17357; RRID:Addgene 17357) [52]. The human Flag-tagged hNaa60 expression plasmid (Flag-hNaa60) was generated by VectorBuilder. The pcDNA-His6-SUMO2(Q90P) mutant plasmid was generated by site-directed mutagenesis using pcDNA-His6-SUMO2 as a template and the following oligos: SUMO2Q90PF-5′ GACGTGTTCCAGCAGCCGACGGGAGGTTAG 3′ and SUMO2Q90PR-5′ CTAACCTCCCGTCGGCTGCTGGAACACGTC 3′.

The following antibodies were used: Anti-HA (BioLegend, #901502) and anti-GFP (BioLegend, #902601), anti-NEDD8 (Abcam, #2745S), anti-p14ARF (Cell Signaling, #2407S and #74560S), anti-UBC9 (Cell Signaling, #4786S), anti-SUMO2 (Cell Signaling, #4971S), anti-SUMO2 (8A2, Hybridoma Bank), anti-beta actin (Santa Cruz Biotechnology, #sc-47778), and anti-glyceraldehyde 3-phosphate dehydrogenase (GAPDH) (Santa Cruz Biotechnology, #sc-32233).

### In vitro SUMOylation assays

In vitro SUMO conjugation assays were performed on [^35^S] methionine labeled in vitro-transcribed/translated proteins, using recombinant SUMO-activating enzyme E1 (Biomol, Lausen, Switzerland), UBC9, and SUMO1 or SUMO2, as previously described [53]. The in vitro transcription/translation of proteins was performed by using 1 μg plasmid DNA and a rabbit reticulocyte coupled transcription/translation system, according to the instructions provided by the manufacturer (Promega, Madrid, Spain).

### In vitro deSUMOylation assay

In vitro deSUMOylation assay was performed on SUMOylated p14ARF protein and using recombinant Sentrin-specific protease 1 (SENP1; Biomol) as previously described [53].

### Purification of histidine-tagged proteins

The purification of His-tagged conjugates using Ni^2+^-nitrilotriacetic acid-agarose beads was performed as previously described [54].

### Endogenous SUMOylation

Analysis of endogenous SUMOylation using denaturing immunoprecipitation was performed as previously described [31]. Briefly, cells were lysed under denaturing conditions and then diluted in non-denaturing buffer and subjected to IP using anti-SUMO2 antibody. Analysis of endogenous SUMOylation using SUBES was performed as previously described [30]. Briefly, total protein extracts were extracted in SUBES buffer (50 mM Tris pH 8.5, 150 mM NaCl, 5 mM EDTA, 1% Igepal) supplemented with protease inhibitor cocktail and 50 μM PR-619 ubiquitin-like proteases inhibitor. One-tenth of input was saved, and the rest of the protein extract was added to a mixture of 50 μl of glutathione-agarose beads and 50 μg of GST-tagged SUBES or the GST control supplemented with 1 mM DTT for 2 h at 4 °C. Bound proteins were recovered by centrifugation. Beads were then washed using 30 column volumes of SUBES buffer. Finally, beads were resuspended in Laemmli buffer, samples were boiled, and the supernatant was analyzed by western-blotting. SUMOylated p14ARF was detected by western-blot analysis using anti-p14ARF antibody.

### Immunofluorescence staining

Immunofluorescence staining, and confocal analysis were performed as previously described [54]. Anti-p14ARF and anti-SUMO2 antibodies were used at a dilution of 1:200. Donkey anti-mouse conjugated to Alexa Fluor 488, and goat anti-rabbit conjugated to Alexa Fluor 594 were obtained from Thermo Fisher Scientific. Analysis of the samples was carried out on a Leica TCS SP5 confocal laser microscope using simultaneous scans to avoid shift between the optical channels. Images were prepared using Adobe Photoshop (Adobe Systems, San Jose, CA, USA).

### Cell cycle analysis

PC3 or PC3-shp14ARF cells were treated with MLN4924 (1 μM). Seventy-two hours after treatment, cells were fixed, permeabilized, and stained in a solution containing propidium iodide, RNase A, and Triton X-100. Then, cells were analyzed for DNA content using a FACS Calibur flow cytometer (BD Biosciences, San Jose, CA, USA) using FlowJo software (FlowJo, Ashland, OR, USA).

### RNA extraction and RT-qPCR assay

Cells were treated with MLN4924 (1 μM) for the indicated time. Total RNA was extracted using GenElute™ Total RNA Purification Kit (Sigma), following the manufacturer’s instructions. cDNA was synthesized using high-capacity cDNA reverse transcription kit (Thermo Fisher), and qPCR was conducted using SYBR Green PCR Master Mix and analyzed using QuantStudio 3 qPCR System (Thermo Fisher) and the primers listed in Table 1.

**Table 1:** Oligonucleotides for RT-PCR.

GAPDH-qRT-F	5′ GGA GCG AGA TCC CTC CAA AAT 3′
GAPDH-qRT-R	5′ GGC TGT TGT CAT ACT TCT CAT GG 3′
SUMO1-qRT-F	5′ AAT TCA TTG GAA CAC CCT GTC TT 3′
SUMO1-qRT-R	5′ AAT TCA TTG GAA CAC CCT GTC TT 3′
SUMO2-qRT-F	5′ AAG ATT AAG AGG CAT ACA CCA C 3′
SUMO2-qRT-R	5′ TCA TCC TCC ATT TCC AAC TG 3′
Ubc9-qRT-F	5′ AAA AAT CCC GAT GGC ACG ATG 3′
Ubc9-qRT-R	5′ CTT CCC ACG GAG TCC CTT TC 3′

### MTT assay

Cells were seeded in 96-well plates in quintuplicate (3000 cells per well) and treated with 1 μM MLN4924 for different times. The growth inhibition effect of MLN4924 was determined using the 3-(4,5-dimethylthiazol-2-yl)-2,5-diphenil tetrasodium bromide assay (MTT) according to the manufacturer’s instruction.

### Caspase 3 staining

Caspase 3 staining was evaluated using the PE Active Caspase 3 Apoptosis kit (BD Pharmigen), following the manufacturer’s instructions.

### RNA sequencing

RNA sequencing was carried out by BGI (Hong Kong). Total RNA was isolated using RNeasy Mini kit. The RNA integrity was assessed using Agilent 2100 Bioanalyzer. RNA-seq libraries were prepared by BGI. RNA sequencing data were analyzed by using Qiagen CLC Genomics Workbench (GWB) v23.0.5. Sequencing reads were first adapters trimmed with GWB “Trim Reads” tool then mapped to Human hg38 genome with default setting. Gene expression was quantified based on annotation Ensembl GRCh38.112. After that, DESeq2 [55] was applied for differential gene expression analysis. The differentially expressed genes used in the heatmap were chosen based on the following criteria: adjusted *p* value i.e., *p*adj < 0.05 and log2FoldChange > 2 or log2FoldChange < −2. The heatmap is generated using the Heatmap function from R package ComplexHeatmap [56]. Expression of each gene (i.e., row) in the heatmap is shown as *Z*-score across all the samples (i.e., columns). GSEA was done by R package “fgsea” [57] on MSigDB Hallmark Pathways (h.all.v2023.1.Hs.symbols.gmt downloaded from https://www.gsea-msigdb.org/gsea/msigdb).

### Statistical analysis

Two-tailed Student’s *t*-tests were used to compare the difference between two groups. One-way ANOVA with Tukey´s correction was used for multiple comparisons. The significance level chosen for the statistical analysis was *P*, 0.05.

## Discussion

Here we demonstrate that p14ARF can be SUMOylated in vitro, in transfected cells and under endogenous conditions, in agreement with the proteomic data that previously identified p14ARF as a potential SUMO substrate [28]. The detection of more than one band corresponding to SUMOylated p14ARF protein in the in vitro or in vivo SUMOylation assays may respond to the presence of more than one SUMO acceptor site in p14ARF or it may be the result of the formation of SUMO2-chains. Our data showing that acetylation of primary amines inhibits p14ARF SUMOylation in vitro, and that the N-terminal part of p14ARF is essential for its conjugation to SUMO in cells, led us to propose that SUMO may conjugate to the N-terminal amino group of p14ARF. The results showing that blocking the free N-amino group of p14ARF by acetylation abolishes p14ARF SUMOylation, suggest that SUMOylation and acetylation compete at the N-terminus of p14ARF to regulate p14ARF. The existence of several bands therefore implies the generation of SUMO2-chains. Formally, however, we cannot exclude the possibility that additional residues might also be targeted. Although it has been previously shown that SUMO can conjugate to mutants with no lysine residues [4, 27], to our knowledge, this is the first demonstration that a naturally occurring lysine-less protein can be SUMOylated. SUMO conjugates at the N-terminal amino group of cofilin-1 [27].

SUMOylation of p14ARF is abolished by SENP1, supporting that the N-terminal conjugation of SUMO to p14ARF is reversible, as previously proposed for cofilin-1 [27]. However, p14ARF was not found to interact with SAE1/SAE2 [6], suggesting that a physical association between p14ARF and SAE1/2 is not essential for its SUMOylation, in contrast to what has been suggested for cofilin-1 [27].

SUMO can modulate the subcellular localization, stability or activity of the substrates [32]. p14ARF is a nucleolar protein [33, 34] and SUMOylation plays an important role in nucleolar function [58], such as regulating the nucleolar localization of its substrates [3, 4, 59, 60]. We could detect co-localization between p14ARF and SUMO2 in the nucleolus and the nucleoplasm. In addition, we did not observe significant differences in the subcellular localization of p14ARF after modulation of SUMOylation, suggesting that SUMO does not regulate p14ARF subcellular localization. Furthermore, we show that a truncated construct of ARF encompassing just the N-terminal 1–28 residues, that does not localize to the nucleolus [61] was still SUMOylated in cells, indicating that nucleolar localization is not required for SUMOylation. Nucleolar-independent SUMOylation is also supported by the detection of p14ARF SUMOylation upon MLN4924 treatment, a condition that promotes nucleolus to nucleoplasm translocation of p14ARF, likely due to nucleolar disruption [40, 62, 63].

Our data reveal that SUMO2 overexpression significantly increases p14ARF protein levels whereas downmodulation of SUMOylation markedly decreases p14ARF protein levels, without obvious effect on the levels of its mRNA, indicates that SUMO stabilizes the tumor suppressor protein, as confirmed using cycloheximide chase assay. Stabilization of p14ARF by SUMO could explain the higher levels of p14ARF protein previously detected in those cells overexpressing the SUMOylation machinery [28]. p14ARF is very unstable in normal cells, but its degradation is inhibited in cancerous cells [64]. Interestingly, SUMOylation enzymes have been found at higher levels in cancer vs normal cells [65–67]. Our results raise the possibility that these two events may be connected.

The molecular mechanism by which SUMO stabilizes p14ARF is still unclear. It has been proposed that those factors that promote the nucleolar localization of p14ARF such as its interaction with nucleophosmin, protect the tumor suppressor from degradation [20, 34, 68–70]. However, the absence of a relationship between SUMOylation and p14ARF nucleolar localization, leads us to propose that SUMO stabilization of p14ARF by SUMO is not through regulation of its subcellular distribution. Previous studies demonstrated that p14ARF is ubiquitinated at its N-terminus [19, 20] and that this process can be promoted by interaction with the ubiquitin ligase ULF [64, 71] or it can be controlled by CRL2 [21, 22]. We observed that treatment with the NEDDylation inhibitor MLN4924 rescues the degradation of p14ARF induced in response to UBC9 depletion or in response to the SUMOylation inhibitor ML-792, and it also increases p14ARF SUMOylation. It has been previously reported that SUMO and ubiquitin can compete for the same lysine residues on their targets [72]. We hypothesize that SUMO and ubiquitin may compete for conjugation to the N-terminus of p14ARF and that the degradation of p14ARF after SUMOylation downmodulation is mediated by a CRL-dependent ubiquitination.

Upregulation of p14ARF stimulates SUMOylation of numerous proteins [1–3, 5, 6]. Here we show that prolonged treatment with MLN4924 induces the upregulation of UBC9 and SUMO transcripts, an increase in UBC9 protein levels and in global SUMOylation; and that these MLN4924-induced activities are reduced in p14ARF-depleted cells, indicating that p14ARF contributes to SUMOylation upregulation in response to MLN4924 treatment.

The NEDDylation pathway is upregulated in some cancers and its inhibition induces diverse tumor-suppressive biological processes. Targeting NEDDylation is therefore a promising strategy for treating cancer [43, 73–75], and the therapeutic potential of the NEDDylation inhibitor MLN4924 is being evaluated in preclinical and clinical trials for cancer treatment. MLN4924 has many effects including induction of DNA re-replication, apoptosis, autophagy, cell growth inhibition, and regulation of T-cell-mediated inflammatory response. Our data reveal that MLN4924 treatment of the p14ARF-expressing PC3 cells also induces SUMOylation upregulation. Moreover, our results indicate that p14ARF contributes to the upregulation of UBC9, SUMO, and p21 protein levels, the G2/M cell cycle arrest, the induction of apoptosis, and the reduction in cell proliferation induced by MLN4924, suggesting that p14ARF is a mediator of the cytotoxic activity of MLN4924.

In summary, our findings reveal that SUMOylation is a new mechanism increasing p14ARF tumor suppressor protein stability, and that treatment with MLN4924 induces the upregulation of p14ARF protein which facilitates the induction of global SUMOylation and a possible positive feedback loop to further increase its levels. Finally, our data suggest that p14ARF status may be an important factor governing cellular sensitivity to MLN4924.

## Supplementary information

Supplementary Figures 1–3

RNA-seq data

uncropped figures

## Data Availability

The RNA-seq data generated during this study are deposited in Gene Expression Omnibus (GEO) with accession number GE:GSE 278308.
