# Supplementary material for: SUMOylation of the lysine-less tumor suppressor p14ARF counters ubiquitylation-dependent degradation
Source: Cell Death Dis. 2025 Jul 12;16(1):519. doi: 10.1038/s41419-025-07854-z (PMC12255780; doi:10.1038/s41419-025-07854-z)
Supplement: Supplementary file 1 — Supplementary Figures 1–3 [file 41419_2025_7854_MOESM1_ESM.pdf]

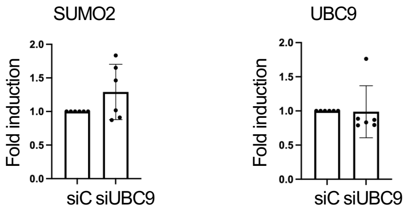

Fig S1. QRT-PCR analysis of PC3 cells transfected with siRNA against UBC9 (siUbc9) or siRNA control (siC). For QRT-PCR analysis, RNA was extracted and expression of mRNAs encoding SUMO2 or Ubc9 were analyzed by QRT-PCR. Values were normalized to GAPDH expression. Columns are representative of the mean and error bars represent the standard deviation of six biological replicas.

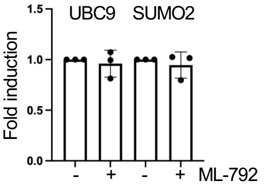

Fig S2. QRT-PCR analysis of PC3 cells treated with DMSO or ML-792. For QRT-PCR analysis, RNA was extracted and expression of mRNAs encoding SUMO2 or Ubc9 were analyzed by QRT-PCR. Values were normalized to GAPDH expression. Columns are representative of the mean and error bars represent the standard deviation of three biological replicas.

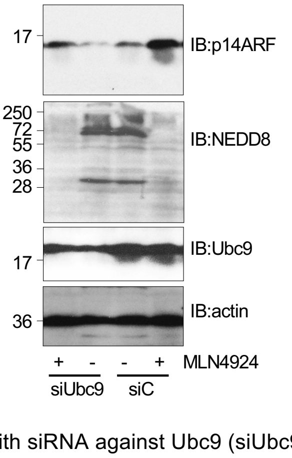

Fig S3. H1299 cells were transfected with siRNA against Ubc9 (siUbc9) or siRNA control (siC) and 48 h after transfection, cells were treated with DMSO or MLN4924. At 48 h after treatment protein extracts were analyzed by western-blot with the indicated antibodies.
