# Supplementary material for: SUMOylation of the lysine-less tumor suppressor p14ARF counters ubiquitylation-dependent degradation
Source: Cell Death Dis. 2025 Jul 12;16(1):519. doi: 10.1038/s41419-025-07854-z (PMC12255780; doi:10.1038/s41419-025-07854-z)
Supplement: Supplementary file 3 — uncropped figures [file 41419_2025_7854_MOESM3_ESM.pdf]

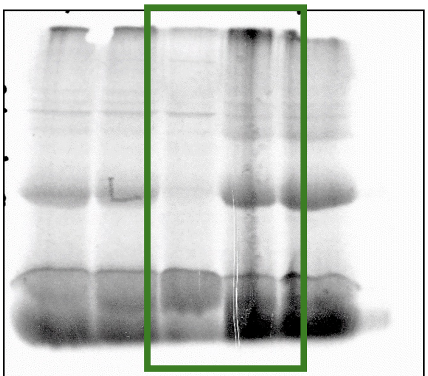

Figure 1A, higher panel

Figure 1A, lower panel

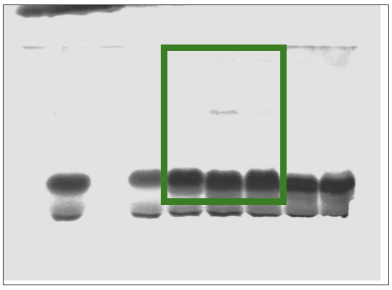

Figure 1B, upper left panel

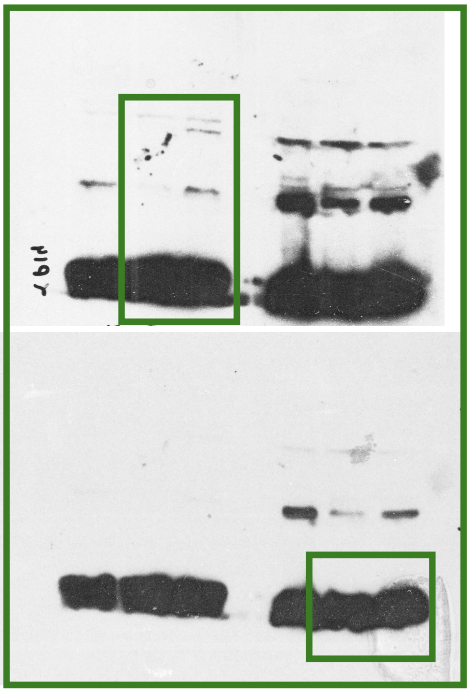

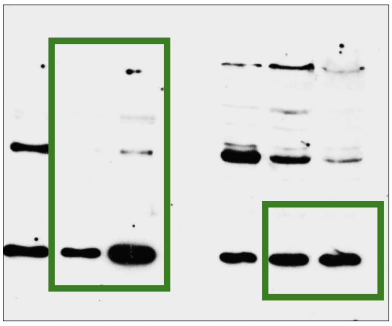

Figure 1B, upper right panel

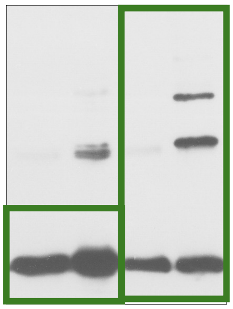

Figure 1B, Lower left panel

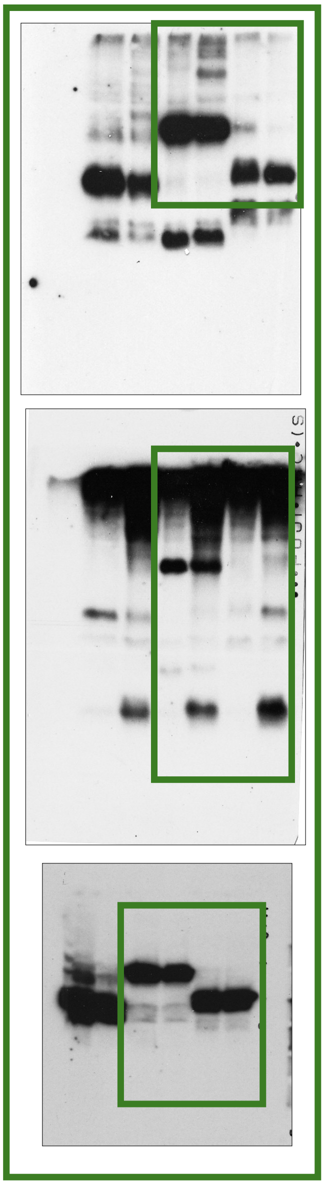

Figure 1B, lower right panel

Figure 1C

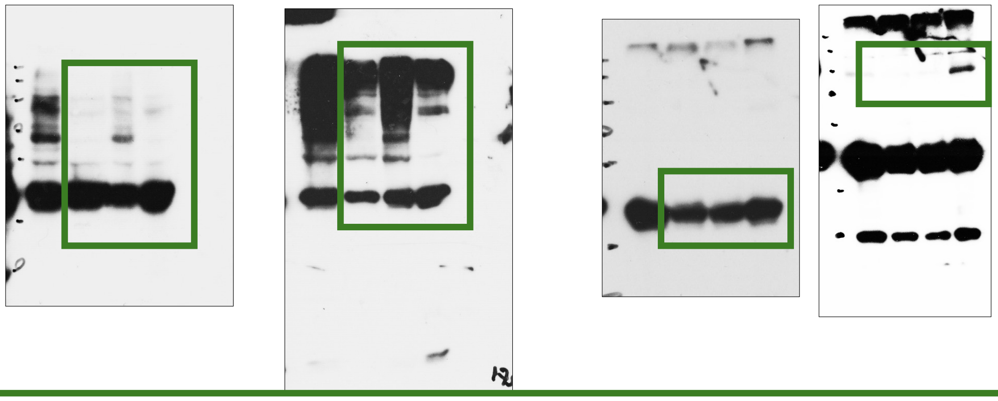

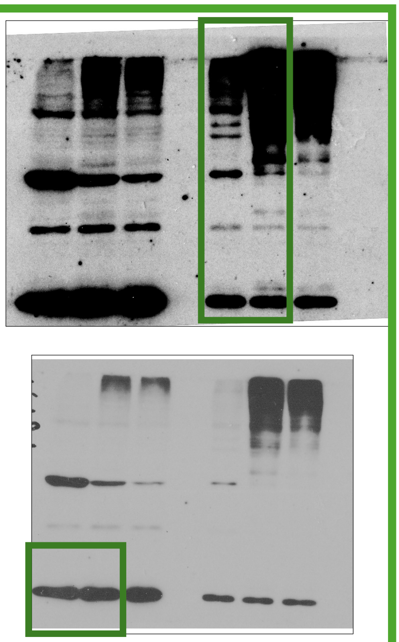

Figure 1D

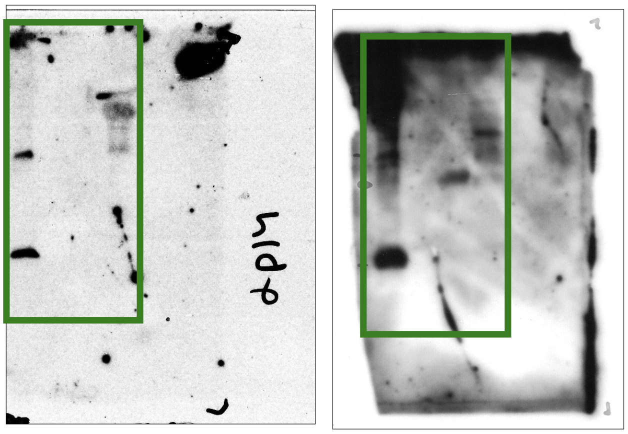

Figure 1D

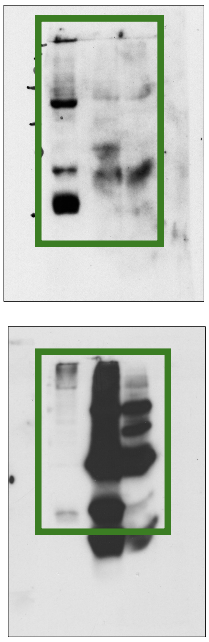

Figure 1E

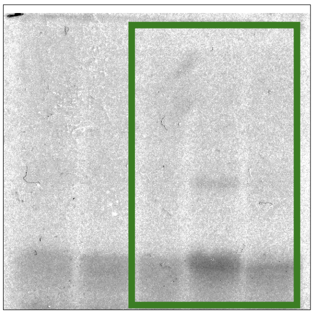

Figure 2A

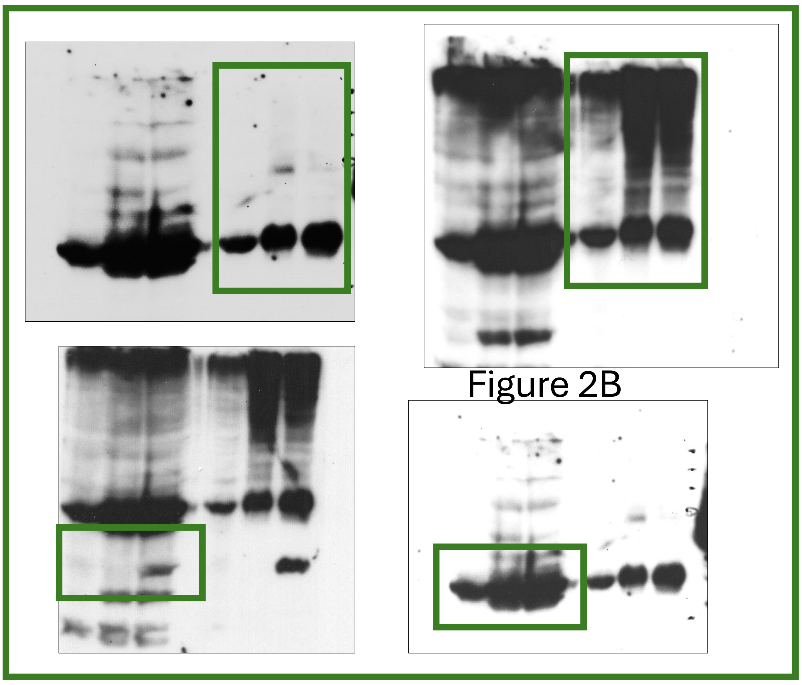

Figure 2B

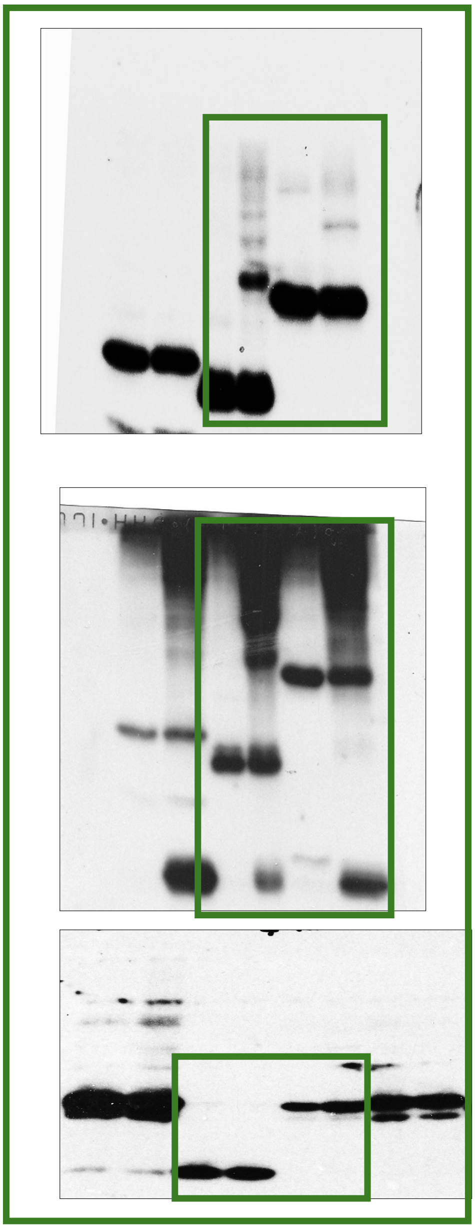

Figure 2D

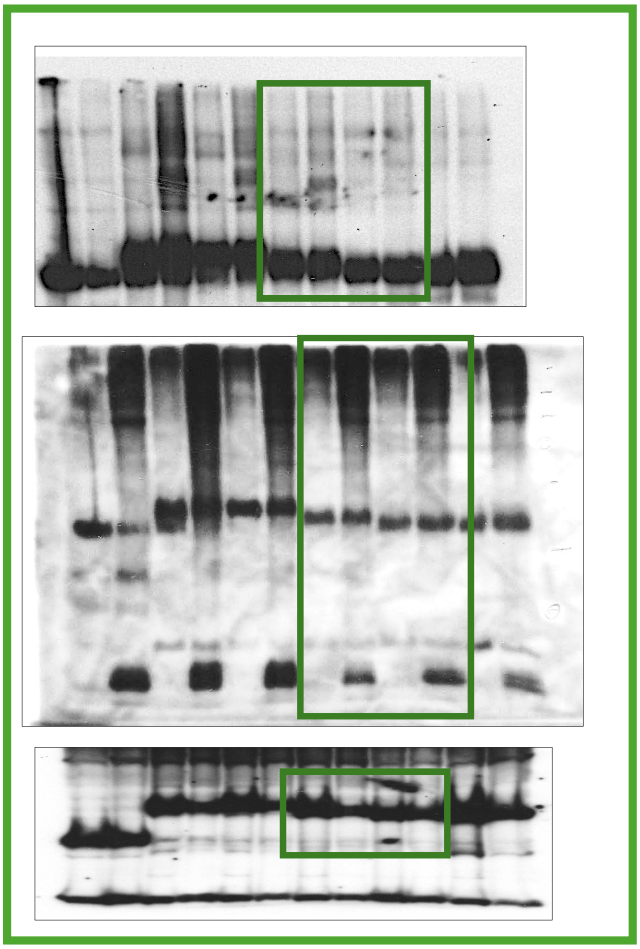

Figure 2E

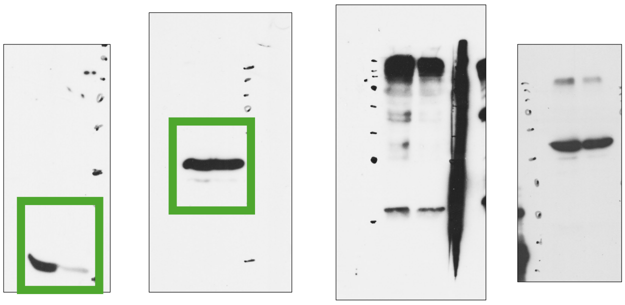

Figure 3A

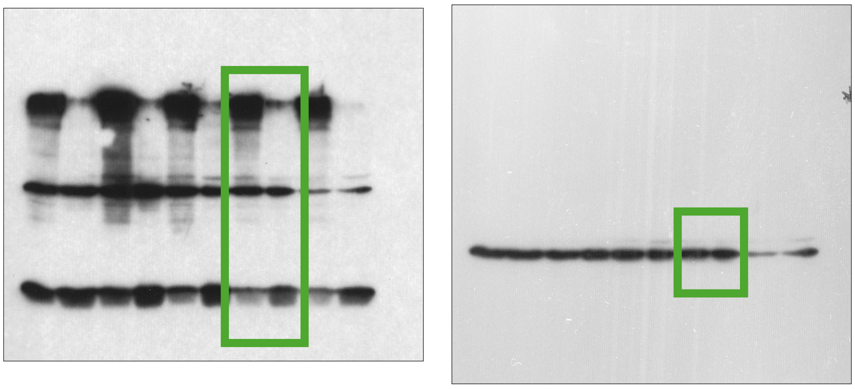

Figure 3B

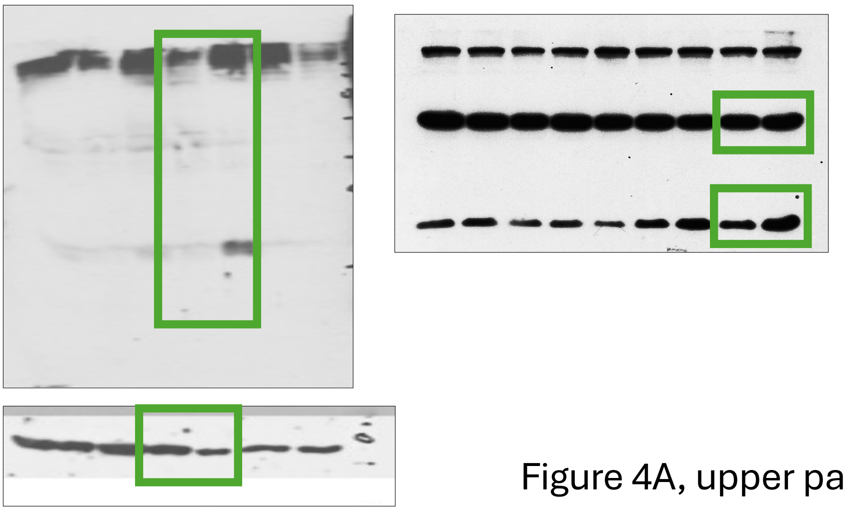

Figure 4A, upper panel

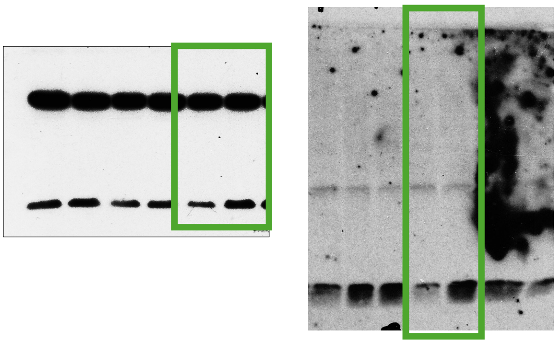

Figure 4A, lower panel

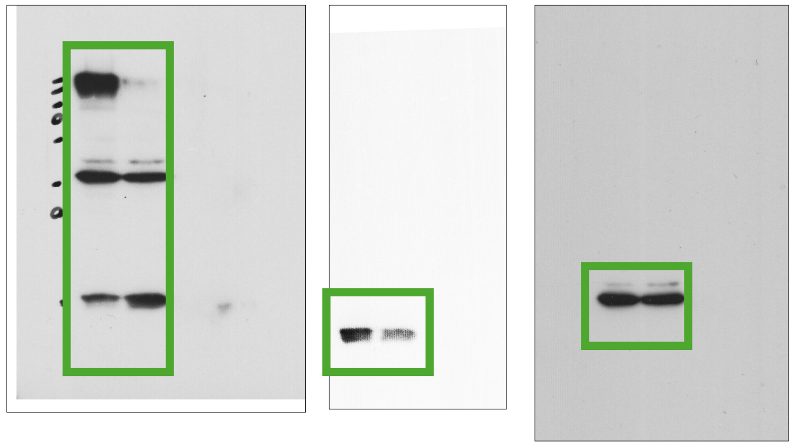

Figure 4B, upper panel

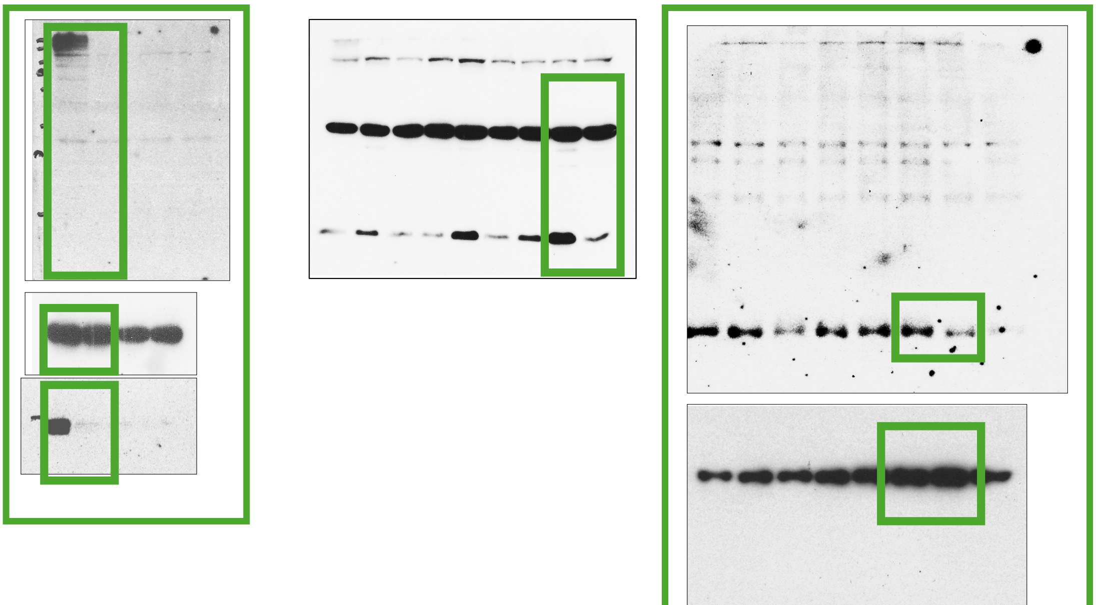

Figure 4B, lower panel

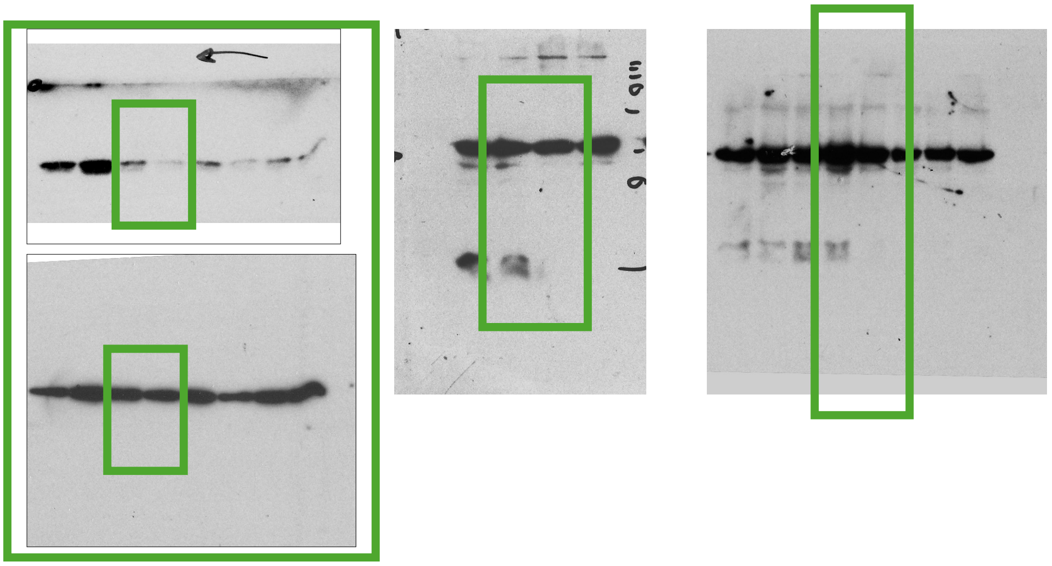

Figure 4C

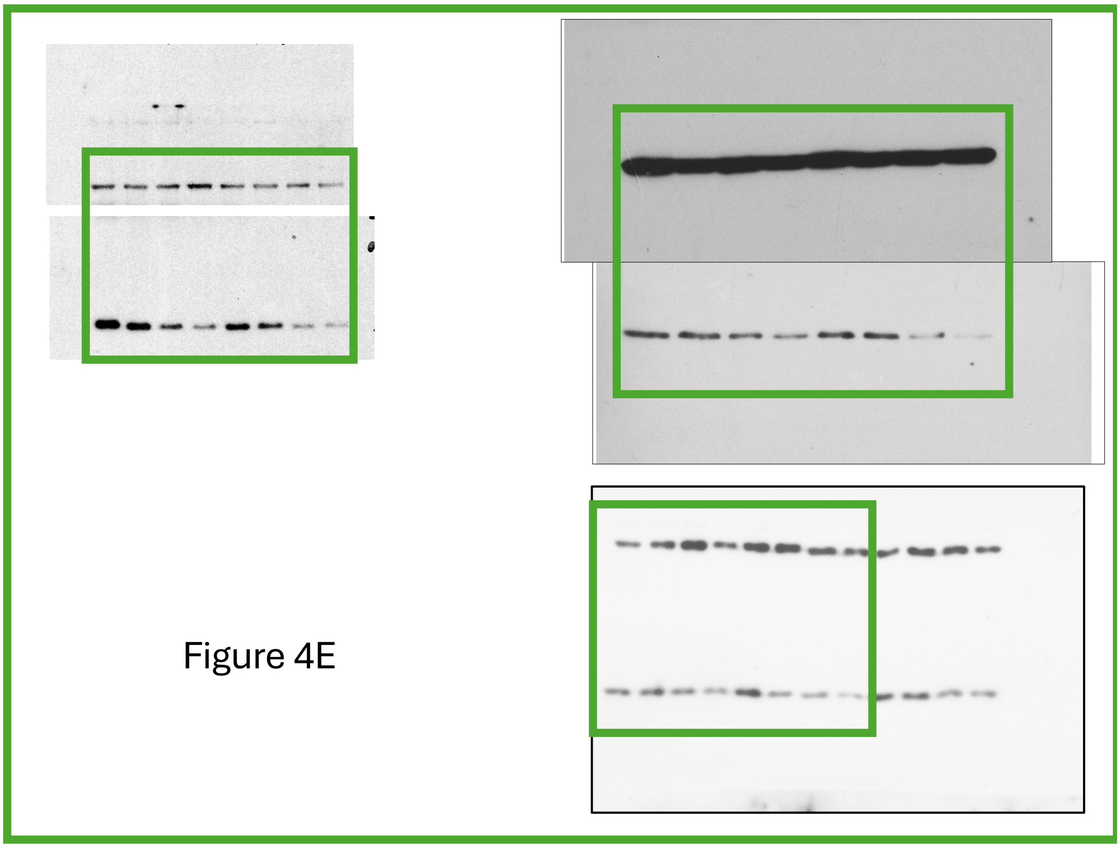

Figure 4E

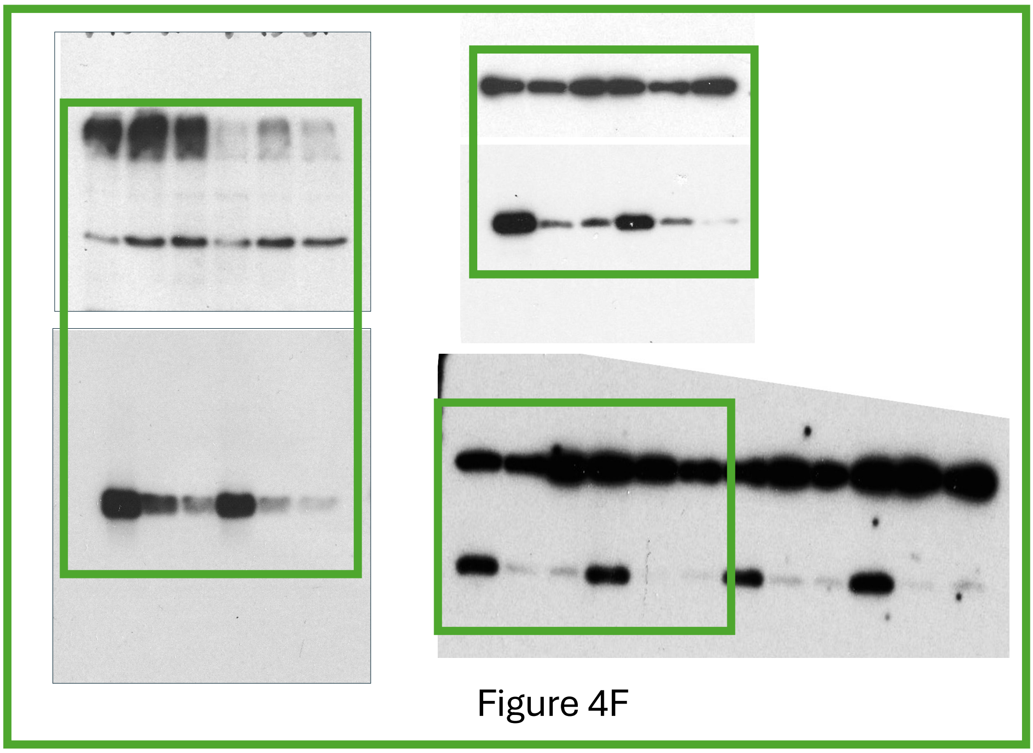

Figure 4F

Figure 5A, upper panel

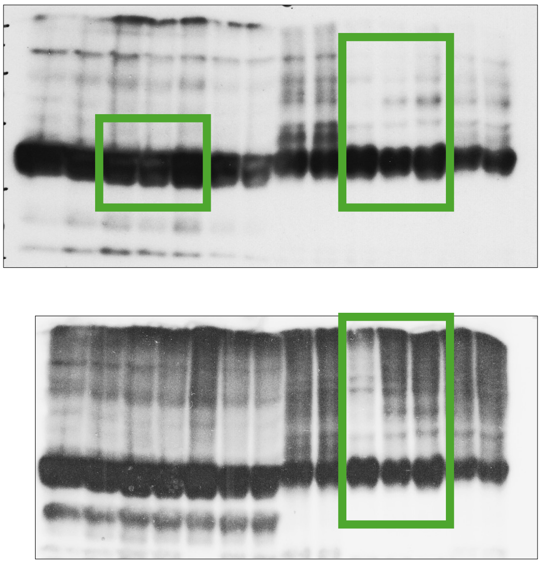

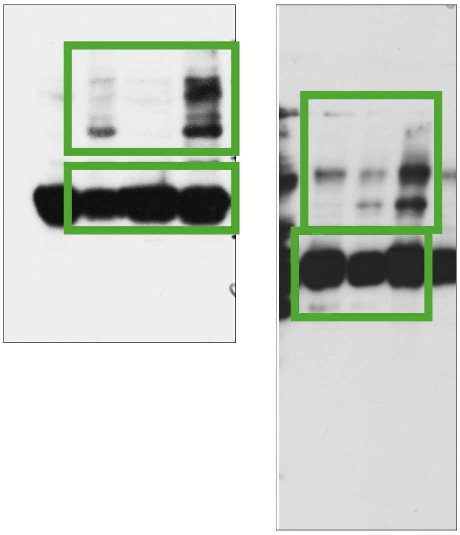

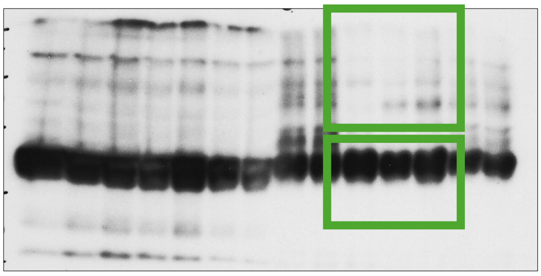

Figure 5A, lower panel

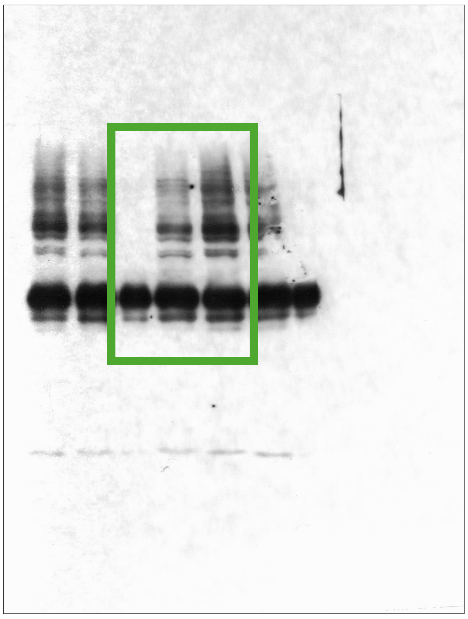

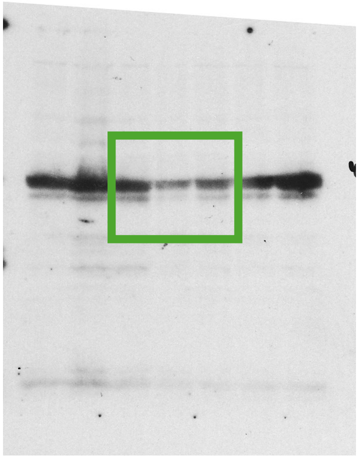

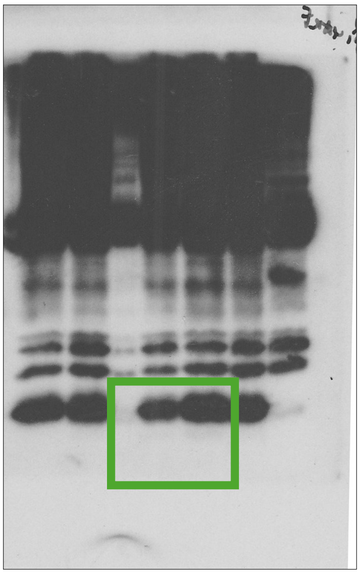

Figure 5B

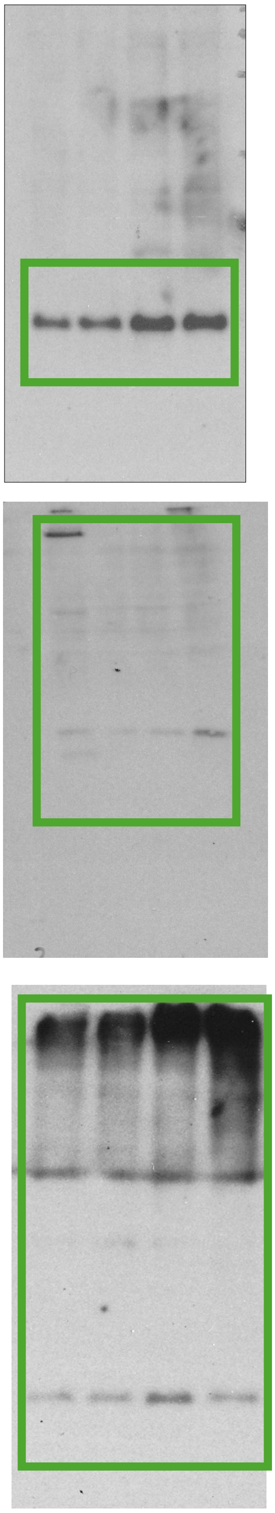
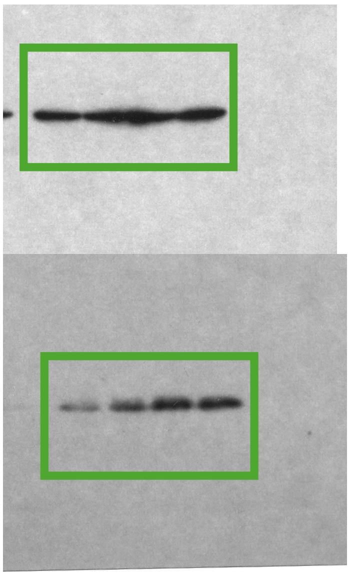

Figure 5C

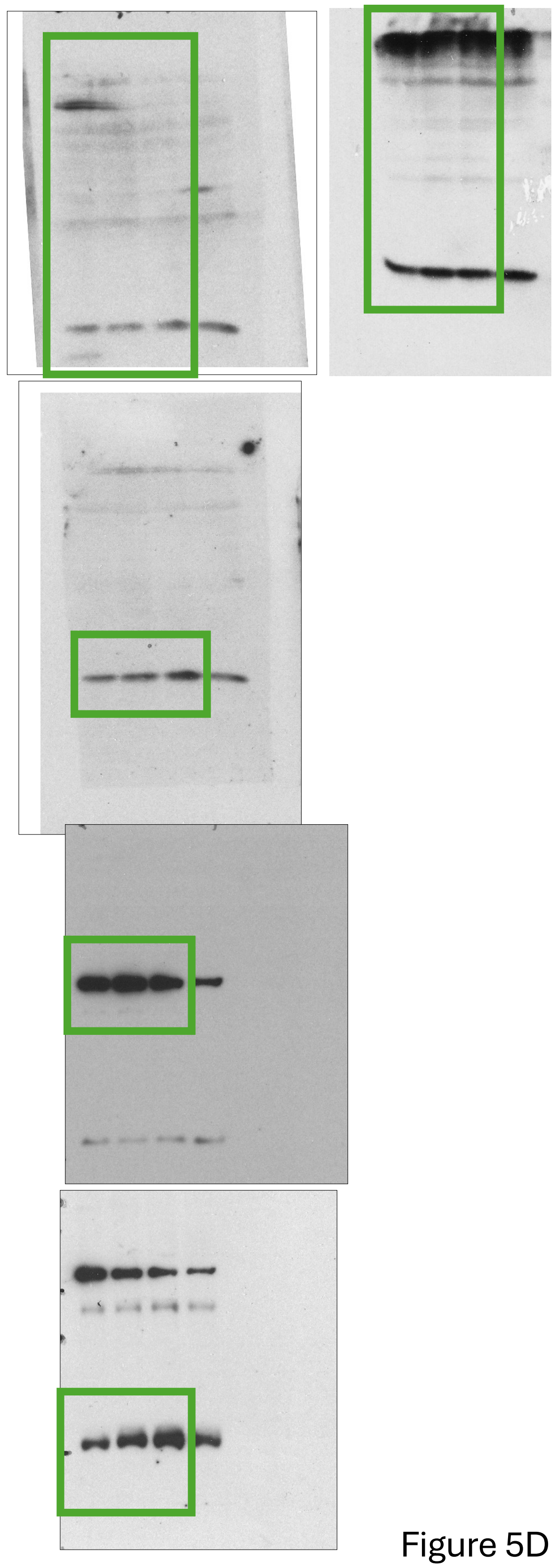

Figure 5D

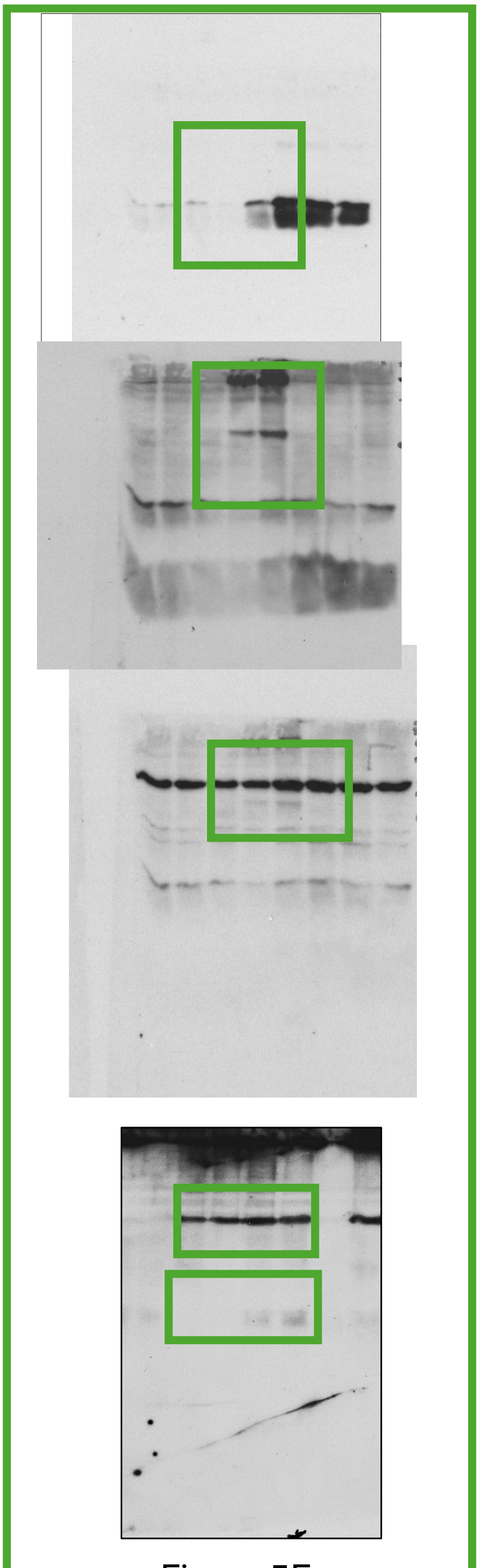

Figure 5E

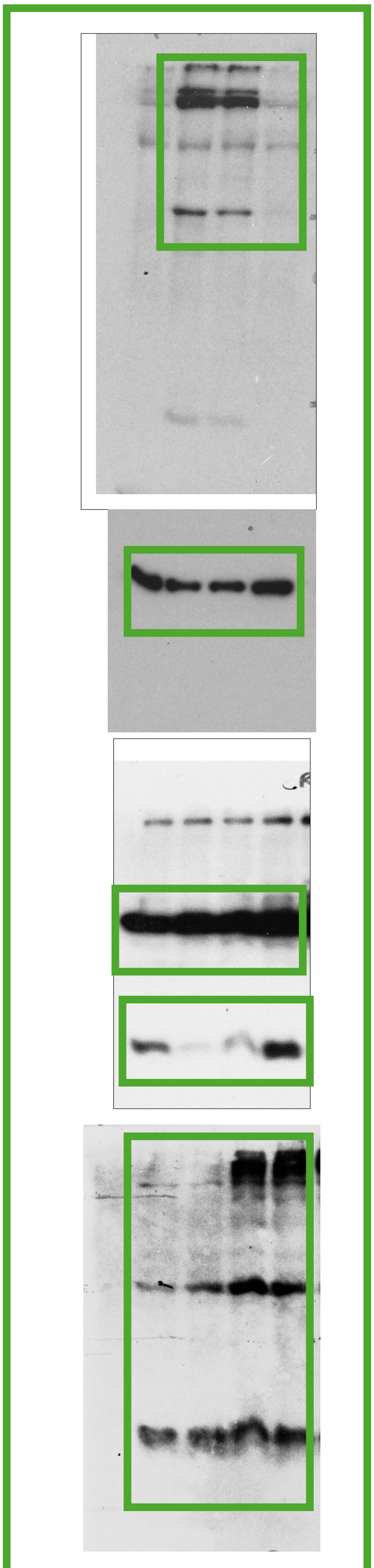

Figure 5F

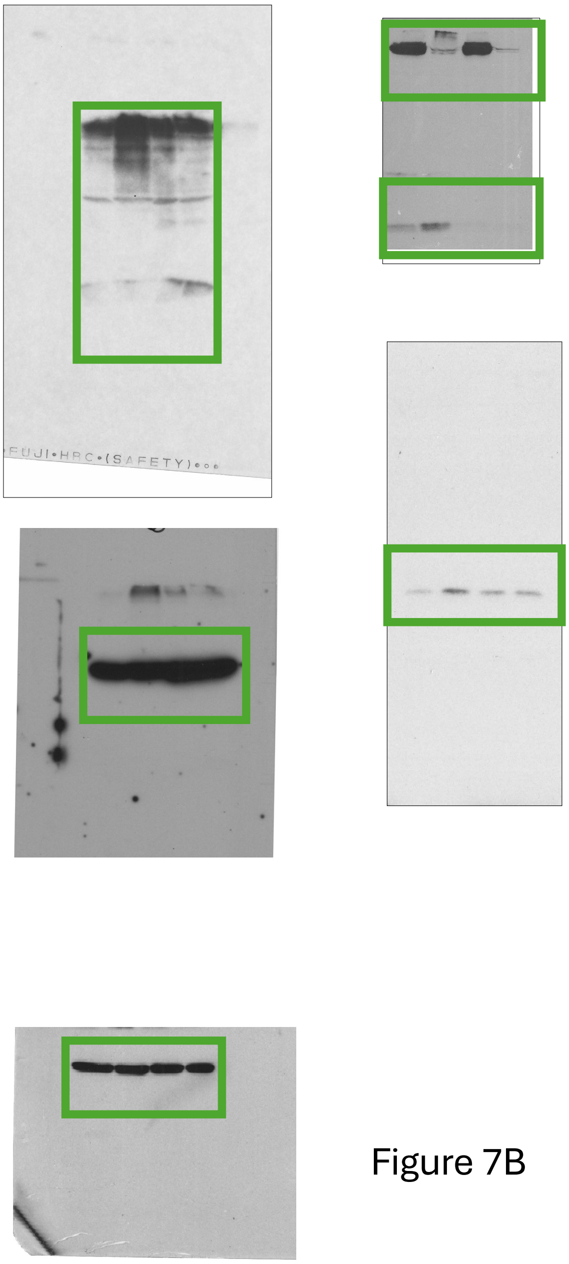

Figure 7B

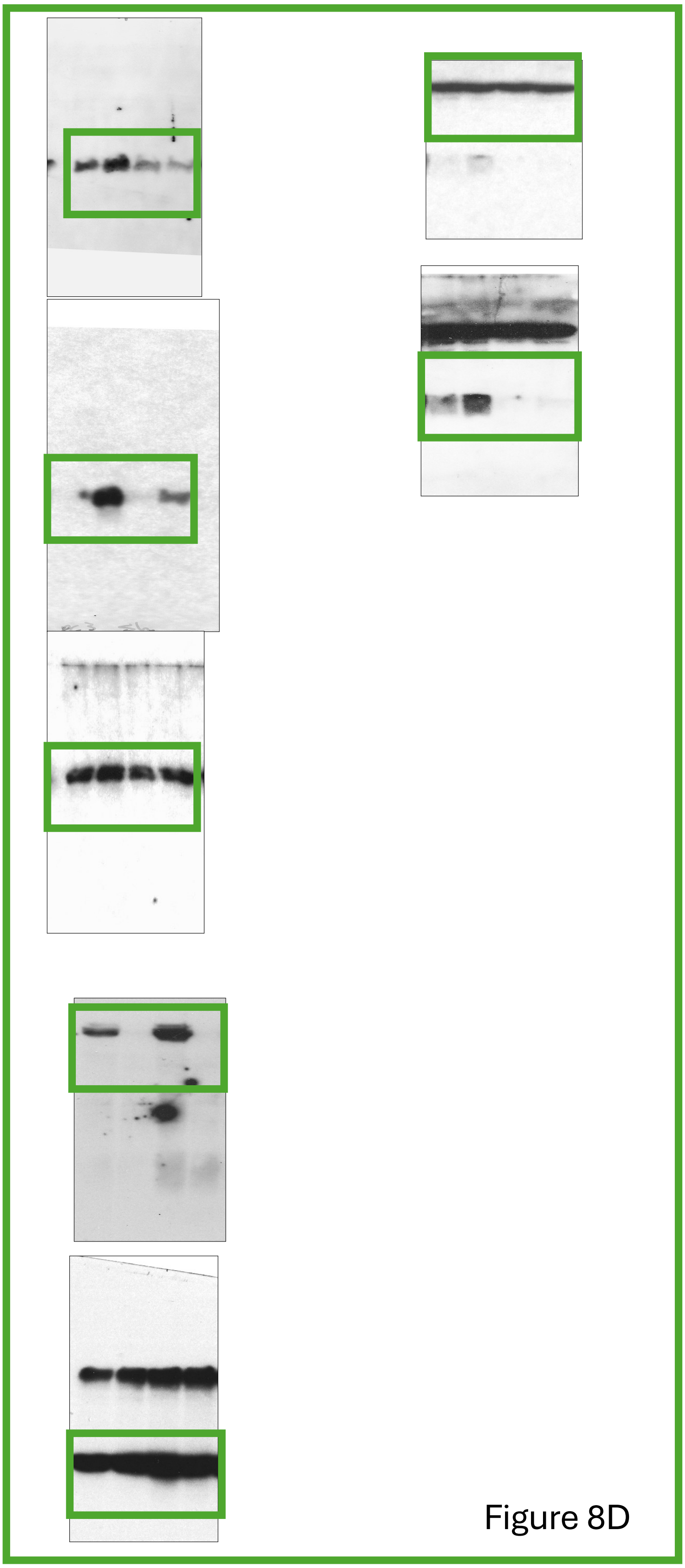

Figure 8D
